# Supplementary material for: ‘I can’t cope with multiple inputs’: a qualitative study of the lived experience of ‘brain fog’ after COVID-19
Source: BMJ Open. 2022 Feb 11;12(2):e056366. doi: 10.1136/bmjopen-2021-056366 (PMC8844964; doi:10.1136/bmjopen-2021-056366)
Supplement: Author's manuscript [file bmjopen-2021-056366.draft_revisions.pdf]

BMJ Open is committed to open peer review. As part of this commitment we make the peer review history of every article we publish publicly available.

When an article is published we post the peer reviewers' comments and the authors' responses online. We also post the versions of the paper that were used during peer review. These are the versions that the peer review comments apply to.

The versions of the paper that follow are the versions that were submitted during the peer review process. They are not the versions of record or the final published versions. They should not be cited or distributed as the published version of this manuscript.

BMJ Open is an open access journal and the full, final, typeset and author-corrected version of record of the manuscript is available on our site with no access controls, subscription charges or pay-per-view fees (http://bmjopen.bmj.com).

If you have any questions on BMJ Open's open peer review process please email info.bmjopen@bmj.com

BMJ Open

“I can’t cope with multiple inputs”: Qualitative study of the lived experience of ‘brain fog’ after Covid-19

Journal:	*BMJ Open*
Manuscript ID	bmjopen-2021-056366
Article Type:	Original research
Date Submitted by the Author:	11-Aug-2021

Complete List of Authors:	Callan, Caitriona ; University of Oxford Nuffield Department of Primary Care Health Sciences<br>ladds, emma; University of Oxford Nuffield Department of Primary Care Health Sciences,<br>Pattinson, Kyle; University of Oxford, Nuffield Department of Clinical Neurosciences<br>Greenhalgh, Trisha; University of Oxford, Nuffield Department of Primary Care Health Sciences<br>Husain, Laiba; University of Oxford, Nuffield Department of Primary Care Health Sciences

Keywords:	COVID-19, INFECTIOUS DISEASES, Infectious disease/HIV < NEUROLOGY, OCCUPATIONAL & INDUSTRIAL MEDICINE, QUALITATIVE RESEARCH

SCHOLARONE™
Manuscripts

I, the Submitting Author has the right to grant and does grant on behalf of all authors of the Work (as defined in the below author licence), an exclusive licence and/or a non-exclusive licence for contributions from authors who are: i) UK Crown employees; ii) where BMJ has agreed a CC-BY licence shall apply, and/or iii) in accordance with the terms applicable for US Federal Government officers or employees acting as part of their official duties; on a worldwide, perpetual, irrevocable, royalty-free basis to BMJ Publishing Group Ltd ("BMJ") its licensees and where the relevant Journal is co-owned by BMJ to the co-owners of the Journal, to publish the Work in this journal and any other BMJ products and to exploit all rights, as set out in our licence.

The Submitting Author accepts and understands that any supply made under these terms is made by BMJ to the Submitting Author unless you are acting as an employee on behalf of your employer or a postgraduate student of an affiliated institution which is paying any applicable article publishing charge ("APC") for Open Access articles. Where the Submitting Author wishes to make the Work available on an Open Access basis (and intends to pay the relevant APC), the terms of reuse of such Open Access shall be governed by a Creative Commons licence – details of these licences and which Creative Commons licence will apply to this Work are set out in our licence referred to above.

Other than as permitted in any relevant BMJ Author's Self Archiving Policies, I confirm this Work has not been accepted for publication elsewhere, is not being considered for publication elsewhere and does not duplicate material already published. I confirm all authors consent to publication of this Work and authorise the granting of this licence.

For peer review only

Original article submitted to BMJ Open August 2021

"I can't cope with multiple inputs": Qualitative study of the lived experience of 'brain fog' after Covid-19

Caitriona Callan[1], academic clinical fellow

Emma Ladds,[1] academic clinical fellow

Laiba Husain,[1] research assistant

Kyle Pattinson[2], clinical professor

Trisha Greenhalgh,[1] clinical professor

[1] Nuffield Department of Primary Care Health Sciences, University of Oxford

[2] Nuffield Department of Clinical Neurosciences, University of Oxford

Corresponding author:

E Ladds: emma.ladds@phc.ox.ac.uk

Nuffield Department of Primary Care Health Sciences

University of Oxford, Oxford OX2 6GG

UK

For peer review only

Key words: Post-acute Covid-19, Chronic Covid-19, long Covid, qualitative study, neuropsychiatric

For peer review only

For peer review only - http://bmjopen.bmj.com/site/about/guidelines.xhtml

Abstract

Objective

To explore the lived experience of 'brain fog'—the wide variety of neurocognitive symptoms that can follow Covid-19.

Design and setting

UK-wide longitudinal qualitative study comprising online interviews and focus groups with email follow-up.

Method

50 participants were recruited from a previous qualitative study of the lived experience of long Covid (n = 23) and online support groups for people with persistent neurological problems following Covid-19 (n = 27). In remotely-held focus groups, participants were invited to describe their cognitive symptoms and comment on others' accounts. Individuals were followed up by email 4-6 months later. Data were audiotaped, transcribed, anonymised and coded in NVIVO. They were analysed by an interdisciplinary team with expertise in general practice, clinical neuroscience, the sociology of chronic illness and service delivery, and checked by three people with lived experience of brain fog.

Results

84% of participants were female and 60% were White British ethnicity. Most had never been hospitalised for Covid-19. Qualitative analysis revealed the following themes: mixed views on the appropriateness of the term 'brain fog'; rich descriptions of the experience of

For peer review only

neurocognitive impairments (especially executive function, attention, memory and language), accounts of how the illness fluctuated—and in some but not all cases, resolved—over time; the profound psychosocial impact of the condition on relationships, personal and professional identity; self-perceptions of guilt, shame and stigma; strategies used for self-management; challenges accessing and navigating the healthcare system; and participants' search for physical mechanisms to explain their symptoms.

Conclusion

These qualitative findings complement research into the epidemiology and underlying pathophysiological mechanisms for neurological symptoms after Covid-19. Services for such patients should include: an ongoing therapeutic relationship with a clinician who engages with the illness in its personal, social and occupational context as well as specialist services that are accessible, easily navigable, comprehensive, and interdisciplinary.

For peer review only

Summary

Strengths and Limitations of Study

- To our knowledge, this is the largest and most in-depth qualitative study of the lived experience of brain fog in survivors of Covid-19.
- The research team was interdisciplinary and interprofessional, and included consultation with patient experts by experience, who helped with data interpretation and peer review.
- Oversampling from men and non-white ethnic groups allowed partial correction of an initially skewed sample.
- The sample was drawn entirely from the UK
- Residual skews in the samples, particularly regarding minority ethnic groups and occupational classes, limited our ability to capture the full range of experiences

Funding statement

This research is funded from the following sources: National Institute for Health Research (BRC-1215-20008), ESRC (ES/V010069/1), and Wellcome Trust (WT104830MA). Funders had no say in the planning and execution of the study or writing up of the paper. KTSP is supported by the National Institute for Health Research Biomedical Research Centre based at Oxford University Hospitals NHS Foundation Trust and the University of Oxford.

Competing Interests Statement

EL and TG provided evidence on long Covid for House of Lords Select Committee

For peer review only - http://bmjopen.bmj.com/site/about/guidelines.xhtml

TG was on the oversight group for the long Covid guideline at the National Institute for Health and Clinical Excellence, and at the time of writing is on the UK's National Long Covid Task Force.

KP and CC have no competing interests to declare.

For peer review only

Background

It is now well-established that COVID-19 can cause persistent ill-health beyond the acute infection, with results from a representative sample of the UK population suggesting that approximately 1 in 5 people will still experience symptoms 5 weeks after infection, and almost 1 in 7 after 12 weeks [1]. Just under 1 in 10 individuals are still affected after 1 year [2]. Over half of those with ongoing symptoms - termed 'long Covid' by patients [3] - experience at least some reduction in ability to carry out their everyday activities, and many report being unable to return to work weeks after the initial infection [1, 4]. The growing number of people with chronic and sometimes disabling illness resulting from the COVID-19 pandemic has made it a policy priority to develop services to meet their health needs [5, 6] and associated clinical and occupational guidelines [7].

Long Covid, a "patient-made" term [3] embraces the formally-defined conditions of post-acute Covid-19 syndrome (symptoms persisting between 4 and 12 weeks) and chronic Covid-19 (symptoms beyond 12 weeks) [7]. It is highly heterogenous in nature, with sufferers reporting a wide range of often-fluctuating symptoms amongst which fatigue, breathlessness, chest pain, post-exertional malaise, autonomic nervous system disruption, and cognitive dysfunction [6, 8, 9] are some of the most common. Whilst the underlying pathophysiology remains unclear, persistent viraemia [10], relapse or reinfection [11] inflammatory and immune reactions [12, 13] , deconditioning [14] and psychological factors [15, 16] have all been proposed as contributors. It is likely that in many patients the causative pathways are multifactorial [17].

For peer review only - http://bmjopen.bmj.com/site/about/guidelines.xhtml

Analysis of the health records of almost a quarter of a million Covid-19 survivors revealed that neurological and psychiatric presentations occurred in both hospitalised and non-hospitalised patients, affecting around one-third of patients over the following 6 months with most severely affected people at highest risk [18]. Around one-quarter experienced disturbed mood, especially anxiety, and a small fraction developed more serious problems such as psychosis. Other neurocognitive problems included substance use disorder, insomnia, cerebrovascular events, encephalitis, dementia, and disorders of peripheral nerves, nerve roots or plexus [18]. Surveys and focus groups conducted on online samples of mostly non-hospitalised long Covid patients have identified impairments in attentional processing, short-term memory and executive function, alongside a general, befuddled state termed 'brain fog' [4, 6, 8, 19]. More recently concern has been raised that such effects may also extend to adolescents and children – a group generally considered to be at 'low risk' from Covid-19 infection [20]. A range of possible pathophysiologies have been identified, including direct neuroinvasion [21], viral persistence and chronic inflammation [22], neuronal injury or toxicity and glial activation [21], microvascular injury [23], activation of autoimmune mechanisms [24], and Lewy body production [25] amongst others, with imaging studies demonstrating a differential loss of grey matter in Covid patients in a number of key brain regions [26].

The functional impact of such cognitive impairment is often profound, affecting individuals' abilities to work and carry out normal daily activities, impeding decision making and judgement, and impairing communication and social relationships, though these impacts have rarely been systematically studied. Guidance for those with neuropsychiatric long Covid symptoms suggests that specialists in clinical psychology and psychiatry should be part of the core multidisciplinary team involved in long Covid rehabilitation [7], but these

For peer review only - http://bmjopen.bmj.com/site/about/guidelines.xhtml

recommendations are contested and inconsistently implemented. Developments in treatment approaches, service pathways, and occupational support structures require further knowledge of both the mechanistic aetiologies underlying such symptoms as well as a better understanding of the lived experiences of those who suffer them.

In this study, we sought to answer three key questions: a) what neurocognitive symptoms are experienced by adults with long Covid?; b) how do these symptoms impact on individuals?; and c) how do they deal with them? We also sought to explore whether our current understanding of psychocognitive processes and the pathological effects of the Covid-19 virus could inform potential mechanistic explanations.

Methods

Study design and governance

This study of people with 50 brain fog was an extension of a previous qualitative study on a large sample of 114 people with long COVID using interviews and focus groups, reported previously [6, 27]. Ethical approval was granted from the East Midlands – Leicester Central Research Ethics Committee (IRAS Project ID: 283196; REC ref 20/EM0128) on 4th May 2020 and subsequent amendments. Participants for the original study had been recruited between May and September 2020 from long Covid support groups on Facebook, a social media call (Twitter), and snowballing (where participants were invited to recruit others known to them). To correct skew, we had oversampled from men and minority ethnic groups. In October 2020, prompted partly by participants' own desire to explore brain fog further, we emailed everyone in this original sample of 114 asking for volunteers to join additional focus groups, and 23 agreed. To extend the sample, 27 additional participants were

For peer review only - http://bmjopen.bmj.com/site/about/guidelines.xhtml

recruited from an online support group dedicated to the neurological effects of long Covid. The dataset for the brain fog study thus consisted of selected data from the original interviews with 23 participants plus five new focus groups with the full sample of 50.

Five focus groups were held in October and November 2020; numbers of participants in each group ranged from 10 to 14. Each group had two facilitators who shared the roles of administering and facilitating the group and taking contemporaneous notes. After a brief explanation and affirmation of understanding and consent, participants were invited to tell the story of their neurocognitive symptoms, with conversational prompts to maintain the narrative and elicit information about the impact on an individual's life and any interaction between symptoms [28]. We encouraged the sharing of stories because the story form is particularly useful for identifying issues important to the patient, identifying emotional touch points in an illness journey, and promoting interaction between participants [29]. One person's story may attract another similar or contrasting story, and reactions to a story (laughter, anger, sarcasm) can add to the dataset.

Data management and analysis

Focus groups were audiotaped with consent, transcribed in full, de-identified and entered onto NVIVO software version 12; contemporaneous notes were also entered. Additional material from the original dataset (where people had raised relevant issues) were also included.

In an initial familiarisation phase, sections of text were arranged into nine broad categories:

For peer review only - http://bmjopen.bmj.com/site/about/guidelines.xhtml

1. Naming the phenomenon
2. Neurocognitive symptoms
3. Natural history of neurocognitive symptoms in long Covid
4. Fatiguability, and interplay between neurocognitive and physical symptoms
5. Psychosocial impact of persistent neurocognitive symptoms
6. Guilt, shame and stigma related to Long Covid
7. Self-management
8. Navigating the healthcare system
9. Hypothesising mechanisms

An interim synthesis was produced from early transcripts and progressively refined using the constant comparative method (data from each new transcript were used to add nuance to the existing synthesis) [30]. Finally, to add more descriptive depth, clarify any discrepancies or ambiguities within the existing data and to track progression (and perhaps resolution) of symptoms, we sent each participant a follow-up email between 4 and 6 months after the focus group (i.e. 10-12 months after their original Covid-19 illness). We asked how their long COVID symptoms were progressing generally as well as asking them to describe their neurocognitive symptoms in detail. 20 of the participants responded to this email and this data was integrated into, and helped refine, our final interpretation.

Theoretical framework

Our analysis was informed by three main theoretical lenses.

- First, from a neuroscience perspective in which SARS-COV-2 (the virus responsible for COVID-19) disrupts function in brain and brainstem networks [31] responsible for maintaining body equilibrium (allostasis), adjusting physiological systems

For peer review only - http://bmjopen.bmj.com/site/about/guidelines.xhtml

(homeostasis) and sensing internal bodily signals (interoception). These systems interact closely with brain systems subserving mood, attention (i.e. fatigue) and cognition [32].

- Second, sociological theories of chronic illness, including May's burden of illness theory, which focuses on the (sometimes extensive) work needed by patients to manage their illness and navigate the system [33], biographical perspectives on chronic illness, which emphasise the impact of the illness on identity and the role of storytelling in shaping and rebuilding that identity [34-36]; and stigma (the depiction by both self and others of illness as shameful and—at least to some extent—the fault of the person) [37].
- Third, emotional touchpoints of powerful feelings such as anger, fear, or hope [38] were identified in participants' experiences of healthcare, and experiences engendering strong positive or negative emotions interpreted using theories of good professional practice, including the physician as wise counsel [39], the therapeutic relationship [40] and continuity of care [41].

Patient involvement statement

The study was planned, undertaken, analysed and written in collaboration with people with long Covid. We gave a webinar presentation via teleconference to which all 50 patient participants were invited, where we presented the key findings including the quotes used in this paper. A recording and copy of the presentation was shared with all participants and all were invited to correct any errors or misinterpretations. The draft paper was modified in response to their feedback. In addition, two clinically qualified people with long Covid reviewed a near-final draft of this paper.

For peer review only - http://bmjopen.bmj.com/site/about/guidelines.xhtml
For peer review only

Results

Description of dataset

Details of participants are shown in Table 1. Despite our efforts to balance for gender and ethnicity, the final sample was skewed to 42 of 50 (84%) female and 36 (72%) White. By comparison, long Covid support groups are up to 86% female [4] and the UK population is 80-85% White British (depending on how defined) [42]. The 5 focus groups, chat transcripts, and follow-up email communications produced over 1000 pages of transcripts and notes. The nine emergent coding themes are discussed in more detail below with illustrative quotes in Table 2 and definitions of neurocognitive processes/functions in box 1.

Naming the phenomenon

Participants varied in their attitudes towards the patient-made term 'brain fog' [4]. Some found it useful as an accessible shorthand to disclose their wide-ranging cognitive difficulties to others, but others felt the term lacked specificity or did not adequately convey the severity of their symptoms (Quote 1).

Neurocognitive symptoms

Participants' description of the symptoms and functional impairments of brain fog were often consistent with deficits in specific domains of cognitive function—particularly executive function, attention, memory and language. Deficits in executive function included problems with planning, decision-making, flexibility and working memory (Quote 2), whilst impairments in complex attention included difficulties with selective, sustained attention,

For peer review only - http://bmjopen.bmj.com/site/about/guidelines.xhtml

divided attention, and processing speed (Quote 3), and long-term memory impairments were seen in free recall, cued recall, procedural memory, and autobiographical memory (Quote 4). The specific language deficits experienced by focus group participants varied between individuals, including difficulties with word-finding and fluency, syntax, reading comprehension and writing (Quote 5).

Natural history of neurocognitive symptoms in long Covid

The longitudinal nature of the study allowed us to explore some aspects of progression of the condition. In the email follow-up, a majority of participants reported that their brain fog symptoms had only become evident after their initial acute Covid illness, with the delay of onset ranging from one to four months, and a majority of participants having ongoing but improving brain fog symptoms at time of follow-up. Of those who felt their brain fog had resolved entirely, the range of time to resolution of symptoms after initial acute illness was 6-10 months (note, however, that this study was not designed to identify precise time course). In those whose symptoms of brain fog persisted, however, these tended to fluctuate both throughout the day and also over a timescale of weeks to months, typically, but not invariably, showing gradual long-term improvement (Quote 6).

Fatiguability, and interplay between neurocognitive and physical symptoms

Fatiguability featured prominently, with many participants describing how either physical or mental effort precipitated a decline in their neurocognitive symptoms. There was also clear interplay between physical and cognitive symptoms, with physical fatigue, tachycardia, or breathlessness most frequently described as impacting the latter (Quote 7).

For peer review only - http://bmjopen.bmj.com/site/about/guidelines.xhtml

For peer review only

Psychosocial impact of neurocognitive symptoms

Participants described profound psychological and social impacts, notably inability to return to work at their previous functional level or even at all. Participants who had returned to work described how they now had reduced hours or adapted roles (e.g. relying on others to check their work), which were often associated with anxiety about potential risks associated with their mistakes (Quote 3), self-doubt about their own abilities, loss of self-worth and altered identity (both professional and personal), as illustrated by Quote 8.

Guilt, shame and stigma

Participants frequently reported strong emotional responses induced by their symptoms and in others' reactions to them. Guilt and shame were particularly evident and often related to difficulties returning to work or their previous level of function or a lack of understanding from others about these problems (Quotes 9 & 10). Particularly troubling were deficits that were not physically visible to other people, and which in some contexts they felt they had to conceal, such as difficulties with language or memory. Participants also described instances of interpersonal conflict arising from their varying cognitive function (Quote 12).

Self-management

Many participants had developed coping strategies to deal with their symptoms, principally around lowering self-expectations and prioritising rest. This resulted in complex self-negotiations and activity trade-offs, including limiting return to work, which participants found frustrating and psychologically draining (Quote 11). Moreover, communication of their reduced, and often varying, cognitive function to family, friends or

For peer review only - http://bmjopen.bmj.com/site/about/guidelines.xhtml

colleagues, was a significant challenge, thus some participants had developed innovative strategies to try and convey their current symptoms and level of functioning (Quote 12)

Navigating the healthcare system

Participants had varying experiences of navigating systems of healthcare, with neurocognitive symptoms often adding to the difficulties of communicating and self-advocating with healthcare professionals (Quote 13). Many described strong negative emotions of frustration, anger and hopelessness associated when they perceived healthcare professionals as having dismissed their symptoms as 'in your head', secondary to depression or anxiety, or not real. Conversely, some participants described a sense of huge relief and validation at feeling believed and having their symptoms acknowledged - often framed as a small victory in the overall uncertainty of long Covid (Quote 14). This was particularly true in the context of interactions with healthcare practitioners, where continuity, wise counselling, and bearing witness were also heralded as desirable components of effective therapeutic relationships (Quote 15).

Hypothesising mechanisms

Participants frequently attempted to make sense of their symptoms and communicate the severity and legitimacy of their suffering through analogous referral to disorders such as stroke, concussion or dementia (Quotes 14 & 16). Many had undergone investigations without identifying a clear cause; in such cases in particular, participants were keen to hypothesise about the physical or neuropsychiatric mechanisms for their as yet unexplained symptoms. Some reported trialling various strategies of self-management, sometimes based on hypothetical mechanisms of long Covid they had read about. These included: dietary

For peer review only - http://bmjopen.bmj.com/site/about/guidelines.xhtml

adaptations – eg: low histamine trials, food supplements eg: zinc, or complementary therapies eg: cannabinoid oils, and were met with varying success.

Discussion

Summary of key findings

This qualitative study of 50 people in the UK suffering from neurocognitive symptoms (brain fog) following Covid-19 has revealed several important findings. Common symptoms in this sample included deficits in executive function, attention, memory and language, which may not be seen – or noticed – until several weeks to months after the acute viral illness, and in most cases followed relapsing-remitting course generally with gradual improvement over several months. Prominent fatiguability and interaction between cognitive and physical symptoms combined with the psychosocial impact on professional and personal activities to produce a destabilising, debilitating, frustrating, stigmatising and frightening situation, impairing individuals' functional ability and damaging their personal and professional identity. They used various approaches to mitigate the effects of brain fog including activity trade-offs and communication strategies, but despite this had only limited success. The experience of illness was greatly compounded by the challenges experienced in navigating the healthcare system—a task which required the very neurocognitive skills they currently lacked.

Comparison with theoretical literature

Some accounts of the varied, uncertain and non-linear nature of this condition fitted Frank's definition of the 'chaos narrative', where the illness experience is unresolved by restitution of

For peer review only - http://bmjopen.bmj.com/site/about/guidelines.xhtml

the former healthy self, thus remains confusing and lacking in meaning [36]. The profound impact of symptoms on individuals' independence, self-efficacy, and self-trust resonated with previous descriptions of spoiled identity and the disrupted sense of purpose and self that can accompany chronic illness [43]. Some narratives also aligned with theoretical accounts of shame and blame in other partly-invisible conditions such as epilepsy [44].

More generally, participants' concerns reflect the well-described phenomenon of 'hidden disability', which requires individuals to undergo a contextual negotiation about when to 'pass' as able-bodied, and when to self-identify as having a disability. In so doing they must weigh up conflicting drivers of self-identity and preservation of self, impression management, stigma, and legitimization of or possible value judgements based on illness-related behaviour [45, 46]. Moreover, the relapsing-remitting time course of brain fog symptoms also align with 'episodic disability', developed by people living with HIV to describe their experiences of unpredictable periods of wellness and illness [47], which adds an additional element of uncertainty to patients' continual assessment.

Such requirements emphasize the extensive work which people with long Covid need to do to manage their condition and navigate services, which accord with theories of illness burden [33]. In particular, the communication and cognitive impairments compound the challenge of self-advocacy and system navigation in a healthcare system that has until recently lacked a clearly defined care pathway [6]. Accounts of positive experiences of care described established dimensions of good professional practice: active listening and bearing witness [40, 48]; wise counsel [39] and continuity of the therapeutic relationship [41] that alleviate patients' illness burden and help begin to construct a healing narrative.

For peer review only - http://bmjopen.bmj.com/site/about/guidelines.xhtml

Lack of mechanistic understanding of the pathophysiological cause was a frequent frustration for participants. Ongoing research has hypothesized that neuronal damage during the initial illness secondary to direct viral neurotoxicity [49] or associated neuroinflammation generate a multisystem dysfunction resulting from a loss of central control and generalized peripheral inflammatory response [50]. Such suggestions are supported by pathological evidence of SARS-CoV-2 neurotropism [51] and neuroinflammation [52] combined with animal models of SARS-CoV-2 infection leading to neuroinflammation, intracellular Lewy body formation, or neuronal loss [25, 53]. It has been hypothesized that such processes impacting on vulnerable brain regions could correlate with neurocognitive long Covid symptoms: dysfunction of the brain stem, which is involved in regulation of both respiration and arousal – and thus potentially 'brain fog' - could result in the attentional deficits and disproportionate breathlessness seen in long Covid [54, 55], though this may not be the only explanation for the symptoms described in our empirical data.

Finally, our findings illustrate that whatever the explanation at the molecular and physiological level, the resultant impacts result from – and contribute to – a far wider interplay of psychological, physical and social factors. The clear disruption to an individual's professional self, interpersonal relationships, and overall sense of identity, combined with the impact of a hidden and episodic disability impair sufferers' abilities to achieve a previously anticipated state of 'health', described by Tarlov as 'the capacity, relative to potential and aspirations, for living fully in the social environment' [56]. Given that long Covid seems to be more prevalent amongst individuals of working age or those still in education, or amongst particular occupational 'key worker' groups who were at greatest exposure risk from Covid-19, the potential impact on society is highly significant. Therefore, whilst further work must deepen

For peer review only - http://bmjopen.bmj.com/site/about/guidelines.xhtml

and exploit our mechanistic understanding, commissioners and providers of long Covid services, as well as individual clinicians, must remain cognizant of the disruption to these broader components of health and wellbeing and consider how they may best be mitigated.

Strengths and limitations of the study

To our knowledge, this is the largest and most in-depth qualitative study of neurocognitive symptoms of long Covid published in the academic literature to date. The research team included both clinicians and social scientists. Our participants spanned a wide range of ages, ethnic and social backgrounds, and illness experiences – including, importantly, the under-researched majority who were never hospitalised. The majority of our participants became infected in the first wave of the pandemic, meaning they are among the earliest cohort of patients to experience long Covid, with email follow-up almost 12 months post-infection giving an insight into the natural history of the condition. We oversampled men and people from non-White ethnic groups to partially correct an initially skewed sample. The use of multiple linked sociological theories allowed to produce a rich theorisation of the lived experience of the illness and draw on that theorisation to produce principles and practical proposals for improving services. We included experts by experience (people with

The study does have some limitations. The entirely UK-based sample included a high proportion of people recruited from a support group for those with neurological symptoms (hence, likely to be more severely affected), and was not fully corrected for some demographic skews. In particular we may not have fully captured the perspectives of some minority ethnic groups or diversity in occupational classes. By pragmatically recruiting largely from social media, we may have introduced an element of selection bias. In the time

For peer review only - http://bmjopen.bmj.com/site/about/guidelines.xhtml
For peer review only

since the first pandemic wave, knowledge and treatment of both acute and long Covid have altered substantially with medical research and patient advocacy (although with geographic variation, and thus inequality, in provision of and access to long Covid services in the UK), which may influence the experience of long Covid for people infected at later time points. It is likely that despite striving do democratic collaborative research *with* patients, we may not have fully grasped the lived experience or represented all voices.

Comparison with previous empirical studies

Our findings of persistent, debilitating neurocognitive symptoms in people with long Covid are in alignment with several retrospective cohort studies [18] and online patient surveys [4, 8, 57, 58]. Our study adds further context to explore the functional and psychosocial impact of such symptoms, their interaction with physical symptoms, and mitigating efforts by patients.

Comparisons have been made between long Covid and other syndromes with neurocognitive dysfunction. Infection with SARS-CoV-1 [59], Epstein-Barr Virus, Coxiella burnetii, Ross River virus [60], and Borrelia burgdoferi [61] can result in similar impairments to concentration and memory, typically correlated with persistent fatigue. However, the challenge of unpicking the underlying aetiology of such symptoms is illustrated by the example of chronic fatigue syndrome/myalgic encephalomyelitis (CFS/ME), where difficulties with executive function, short-term memory, attention and word-finding are incorporated in the diagnostic criteria of both the UK National Institutes for Clinical Excellence [62], US Centers for Disease Control and Prevention [63], and International Consensus Group [64], but where the underlying cause remains unclear.

For peer review only - http://bmjopen.bmj.com/site/about/guidelines.xhtml

For peer review only

Moreover, examples such as HIV-associated neurocognitive dysfunction, which afflicts over 40% of people with chronic HIV infection [65], impairing learning, memory, attention, and executive function, suggests possible overlap across multiple chronic viral infections. A recent study in Nature illustrates how such higher order disruptions may be mediated on a molecular level through viral-associated perturbations in general cellular functions such as cortical excitatory synaptic signalling, choroid plexus disruption enabling peripheral T cell infiltration, and promotion of pathological microglial and astrocyte subpopulations [66]. All of these mechanisms – and others – will require further elucidation.

Both the partially hidden nature of the neurological disabilities experienced by long Covid patients and the extensive work required to manage these and navigate services may exacerbate the impact of the epidemiological distribution of persistent symptoms. Recent data from the Office for National Statistics demonstrated that self-reported long Covid was greatest in people aged 35-69 years, women, people living in the most deprived areas, those in health and social care occupations, and those with another activity-limiting health condition or disability [2]. As for the acute infection, long-term sequelae of Covid-19 infection are strongly impacted by socioeconomic determinants such as poverty and structural inequalities such as racism and discrimination [67], which may affect health beliefs, health-seeking behaviours, or the response of health services. Whilst not directly reported by participants in this study, further work to explore the impact of such determinants on long Covid epidemiology and interactions with health services will be crucial to mitigate the impact of associated disability.

For peer review only - http://bmjopen.bmj.com/site/about/guidelines.xhtml

Conclusion: implications for services and further research

In dealing with Covid-19, it is crucial that health policy begins to shift from an acute disaster response to managing a chronic crisis. This study has brought neuroscientists together with qualitative researchers to try to align the subjective illness experience as directly described by patients with the objective disease models that underpin therapeutic options for ongoing 'brain fog' experienced by long Covid patients. The profoundly disabling, persistent impacts in some people revealed here adds weight to arguments that we need to prevent Covid-19 in order to reduce the long-term burden of this disease on patients, the health service, and the wider economy. Moreover, it is crucial to mitigate the impact for those already affected through a better understanding of the pathophysiological mechanisms of this neurotrophic virus and further exploration of the best approaches to support cognitive, psychological, and occupational restoration.

The strong positive and negative emotional touchpoints [38] described by individuals when their accounts are—respectively—believed or dismissed underscores the importance of the clinical relationship in which the patient is listened to, believed, and supported — particularly in primary care, which is likely to be the patient's first point of contact [68, 69]. Furthermore, the varied nature of the severe impacts of brain fog identified in this study highlight the importance of ensuring that specialist services for this condition are accessible, easily navigable, comprehensive and interdisciplinary—for example incorporating (where necessary) assessment and rehabilitation from clinical psychologists and occupational therapists [7]. Our findings affirm those of a previous study (with a partially overlapping sample) to co-design quality indicators for long Covid services, which emphasised the importance of continuity, clinical responsibility, multidisciplinary input, patient involvement, and use of evidence-based guidelines [6].

For peer review only - http://bmjopen.bmj.com/site/about/guidelines.xhtml

For peer review only

Acknowledgements: We thank the 50 participants for their interest and contributions, and two experts by experience for helpful comments on a draft of the paper: Sharon Taylor, child psychiatrist and honorary senior lecturer at the Central and North-West London NHS Foundation Trust and Imperial College School of Medicine and Clare Rayner, Independent Occupational Physician, Manchester. Alex Rushforth and Sietse Wieringa undertook interviews for the original study of long Covid.

Contributors and sources: EL and TG conceptualised and designed the study. EL, LH, CC, and KP conducted focus groups. EL and CC led data analysis, with input from LH, TG and KP and produced a first draft of the results section. EL and CC wrote the first draft of the paper which was refined by all authors. LH provided research assistant support and conducted some interviews. ST and CR provided expertise by experience and knowledge of patient-led research. CC presented findings to long Covid patient participants with assistance from EL and TG. All authors contributed to refinement of the paper provided additional references. EL is corresponding author and guarantor. EL affirms that the manuscript is an honest, accurate, and transparent account of the study being reported; that no important aspects of the study have been omitted; and that any discrepancies from the study as planned (and, if relevant, registered) have been explained.

Copyright: The Corresponding Author has the right to grant on behalf of all authors and does grant on behalf of all authors, a worldwide licence to the Publishers and its licensees in perpetuity, in all forms, formats and media (whether known now or created in the future), to i) publish, reproduce, distribute, display and store the Contribution, ii) translate the Contribution into other languages, create adaptations, reprints, include within collections and

For peer review only

For peer review only - http://bmjopen.bmj.com/site/about/guidelines.xhtml

create summaries, extracts and/or, abstracts of the Contribution, iii) create any other derivative work(s) based on the Contribution, iv) to exploit all subsidiary rights in the Contribution, v) the inclusion of electronic links from the Contribution to third party material where-ever it may be located; and vi) licence any third party to do any or all of the above.

Table 1: Participant characteristics

	Participants recruited from previous long Covid study	Participants recruited from neuro Covid support groups	Total Brain Fog Focus Group Participants	Responders to email follow-up post-focus groups
	23	27	50	20
Gender				
• Female	15	26	42	17
• Male	8	1	8	3
Age				
• Median	48	36	43	43
• Range	31-74	29-68	29-74	31-74
Ethnicity				
• White British	16	14	30	11
• White other	3	3	6	1
• Black	1	1	2	0
• Asian	3	2	5	1
• Mixed	0	0	0	0
• Non-response	0	7	7	7
Occupation				
• Healthcare professional	8	8	16	5
• Non-healthcare professional	13	11	24	9
• Non-response	2	8	10	6
Hospitalised at any point due to Covid-19				
• Yes	0	4	4	4
• No	9	8	17	16
• Non-response	14	15	29	

For peer review only - http://bmjopen.bmj.com/site/about/guidelines.xhtml

Box 1. Definitions

Planning: the mental process allowing individuals to choose necessary actions to reach a goal, ascertain the required order, assign tasks to cognitive resources, and establish a plan of action.

Decision making: the cognitive process resulting in the selection of a belief or a course of action from multiple possible alternative options,

Flexibility: the mental ability to adjust activity and content of the cognitive system i.e. enabling a switch between different task rules and corresponding behavioural responses, maintaining multiple concepts simultaneously and shifting internal attention between them.

Complex attention: a person's ability to maintain information in their mind for a short time and to manipulate that information eg: to perform mental arithmetic calculations.

Selective sustained attention: the ability to focus on an activity or stimulus over a long period of time even if there are other distracting stimuli present.

Divided attention: the ability to attend to multiple different stimuli at the same time, thus responding to more than one demand from the surroundings i.e. enabling multi-tasking.

Processing speed: the time it takes a person to do a mental task i.e. the at which a person can understand and react to the information they receive from sensory inputs and generate a reaction.

Working memory: a cognitive system with a limited capacity, capable of temporarily holding information to enable reasoning and guiding decision-making and behaviour.

Procedural memory: a type of implicit memory that aids the performance of particular types of tasks without conscious awareness of previous experiences eg: stored motor programmes of particular well-rehearsed actions.

Autobiographical memory: a memory system formed from episodes recollected from an individual's life that combines episodic (personal experiences and specific objects, people and events experienced at particular time and place) and semantic (general knowledge and facts about the world) memory.

Free recall: a common memory task requiring individuals to recall any items from a previously memorized list either immediately or following a delay.

For peer review only - http://bmjopen.bmj.com/site/about/guidelines.xhtml

Cued recall: As above, individuals are required to recall items from a previously memorized list but may be given cues (often semantic) to encourage this.

Table 2: Participant Quotes

Identifier	Source	Quote
1	Participant 10, Focus Group (FG) 4	"Does anyone ever refer to it as neurocognitive fatigue? In a way I don't like brain fog as it's too vague, too loose of a term, so want something more technical. Though I don't think neurocognitive fatigue encompass the word finding difficulties, so it's not ideal either"
2	Participant 7, FG1	"One of the things I've realised is how many things I do in my normal day - I'm not talking about work, just in a normal day - that are cognitive that I [didn't previously] think of as being cognitive. So a supermarket, the amount of sensory information, and just staring at a row of things looking for the food that you want, remembering where things are in the aisles and planning your trip so that you don't have to walk backwards and forwards around the shop, that surprised me. […] Not just can I walk around the supermarket, it's planning, it's getting there, it's choosing stuff, all of that is actually really difficult."
3	Participant 5, FG1	"I can't cope with multiple inputs, like if I'm trying to reply to a message on my phone and one of my boys starts speaking to me or there's something else happening as well that just really fries my brain. I mean I used to be the kind of person that, like all

For peer review only

		women, multi-tasking was a superpower. I was able to, do lots and lots of things, you know I'm [a doctor]; I would have one patient I'd be hearing lots about another patient coming I'd be remembering I'd be doing something else I'd be juggling lots and lots of things and now I can't keep multiple plates spinning I absolutely can't. I've got to focus on just one thing or I make massive mistakes and it's like I forget my intentions all the time."
4	Participant 10, FG3	"I can ask somebody a question and then I'll ask the exact same question two minutes after and not remember I've asked them, I can't remember significant things that have happened in the past either"
5	Participant 8, FG2	"[It's difficult] to comprehend and take in written information and read it. I had a form sent to me at work and I just felt, 'I can't do this at the moment' and put it to one side and hoped to come back to it because it's just been too difficult"
6	Participant 3, FG5, in email response to follow-up	"I'm probably about 90% better. I'm struggling to put in full days at work and still need a great deal of rest and sleep. My brain fog is greatly improved, although I'm making mistakes at work and have been forgetful and sometimes confused with large amounts of new information. I feel like my head is clear now. When you did the group interview I felt like I was drugged up all of the time. Now it's far and few days between that I feel that way. I think the brain fog lasted around eight months."
7	Participant 9, FG1	"I've had times when I've tried to do teaching or have meetings via Zoom or just spent a lot of time doing computer work, then I'd

For peer review only

		often relapse the following day, what I get is burning lungs, chest pain, breathlessness and the tachycardia. So without a doubt the mental exertion or the energy required to do the thinking and processing then has a detrimental effect on me physically”
8	Participant 11, FG3	“Seven months plus in I don’t know whether I’m gonna get my brain back […] I’m really, really fearful for the future or whether I’m going to be able to get back to what I want to do and that’s like your identity and yourself and who I am as a person is, you know, a big part of me is being a [allied health professional] and if I can’t, if I’ve lost that, I’ve lost a huge part of me.”
9	Participant 9, FG4	“I found myself restating and reiterating many times professionally where I’m at now in terms of cognitive ability and there’s only so many times you can do that before I feel like I’m becoming that person, you know and it’s a lot easier to do that in the house but I think professionally it’s been really hard”
10	Participant 5, FG4	“a few times that I’ve been out and had an in-depth conversation with somebody that hasn’t managed to get used to how I am, they’ve sort of said to me “you’re going round in circles in your conversation” or “you’re not making a lot of sense”, when I hadn’t quite recognised how repetitive I was being until somebody said it back to me. But even so those same people … can’t seem to cut me any slack for it, or can’t seem to understand how difficult it is, do you know what I mean? [There] just doesn’t seem to be the understanding there and I can understand that because it would be beyond my comprehension as well if I hadn’t lived it”

For peer review only

For peer review only - http://bmjopen.bmj.com/site/about/guidelines.xhtml

11	Participant 5, FG2	"For me it's been going from working at 110% pace to not being able to get out of bed, not being able to work to not see people, to have to cancel plans, the impact on my life has been a massive transition and getting my head around that has been huge. I'm accepting now that I need to take the time off to get better and although that's really difficult and it's meant letting lots of people down, and there's been a complete change in my life, I've managed to get to that place."
12	Participant 7, FG4	"Me and my husband have got a traffic light system now, so green's fine, he can just talk business at me, amber is like can you just keep 'what's the weather'-like kind of conversation, and then red is just stop, I need to just rest, stop all the sensory input coming in. And that seems to be working quite well now, so literally I've got to say amber or red and it's that thing when you're so tired that you can't even articulate that you're so tired and explain. So that really has helped us and I think might stop quite a lot of rows."
13	Participant 2, FG1	"I've gone to the GP's and it's like I speak to someone different every time which is not helpful for that continuity and that consistency and it's like I have to go right back to the beginning and it's almost like I'm not believed that I even had Covid, and it's like I'm so far away from my normal and it's that trickiness of having to re-explain in that ten minutes and then you just go bluurrghh and it like all comes racing out and you're like 'I've

For peer review only

		no idea what I've just said, a) whether it makes sense or b) whether I actually got my point across'"
14	Participant 8, FG1	"I have to say it was when my GP said 'yes, we recognise what you've got as Long Covid and we're treating it like concussion at the moment until we know more about it, and we will recommend you rest and maybe try these drugs', I mean, I almost broke down it was the acknowledgement of the issue. [It] takes away so much of the stress because, we're all [thinking], you know, 'is this really happening, is this just me malingering or do I really have this thing'. And so that was that was a key moment for me"
15	Participant 7, FG 1	"I had a couple of different GPs that I spoke to at the beginning and then I spoke consistently to the same locum GP and she was very good. It was when I was having quite a difficult time trying to go back to work and I was struggling quite a lot psychologically and she was very supportive, she spent a lot of time with me and that consistency was good"
16	Participant 13, FG2	"I've treated stroke patients who [have] dysphasia and they can't find the right words so they go around the houses to describe something so that you understand what they mean and it felt a bit like that in a way that you know what you want to say but you can't think what that word is because it doesn't come to the forefront of your mind. So you're trying to think of how you can describe it and I thought 'oh gosh, I've turned into one of my

For peer review only

For peer review only - http://bmjopen.bmj.com/site/about/guidelines.xhtml

		stroke patients' because I'm trying to find another suitable word but it's such a struggle though"

For peer review only

For peer review only - http://bmjopen.bmj.com/site/about/guidelines.xhtml

References

1 Office of National Statistics. Prevalence of ongoing symptoms following coronavirus (COVID-19) infection in the UK: 1 April 2021. 2021.

2 Office of National Statistics. Prevalence of ongoing symptoms following coronavirus (COVID-19) infection in the UK-1 July 2021
. 2021.

3 Callard F, Perego E. How and why patients made Long Covid, *Social science & medicine (1982)* 2021;268:113426 doi:10.1016/j.socscimed.2020.113426 [published Online First: Jan].

4 Davis HE, Assaf GS, McCorkell L, et al. Characterizing Long COVID in an International Cohort: 7 Months of Symptoms and Their Impact, 2020 doi:10.1101/2020.12.24.20248802.

5 World Health Organization. In the wake of the pandemic: preparing for Long COVID (2021). ;2021.

6 Ladds E, Rushforth A, Wieringa S, et al. Persistent symptoms after Covid-19: qualitative study of 114 "long Covid" patients and draft quality principles for services, *BMC Health Serv Res* 2020;20:1144 doi:10.1186/s12913-020-06001-y [published Online First: -12-20].

7 NICE guideline. COVID-19 rapid guideline: managing the long-term effects of COVID-19. 2020;2021.

8 Ziauddeen N, Gurdasani D, O'Hara M,E., et al. Characteristics of Long Covid: findings from a social media surve, 2021.

9 Sudre CH, Murray B, Varsavsky T, et al. Attributes and predictors of long COVID, *Nature medicine* 2021:1-6 doi:10.1038/s41591-021-01292-y [published Online First: Mar 10,].

10 Wu F, Wang A, Liu M, et al. Neutralizing Antibody Responses to SARS-CoV-2 in a COVID-19 Recovered Patient Cohort and Their Implications. 2020.

11 Lan L, Xu D, Ye G, et al. Positive RT-PCR Test Results in Patients Recovered From COVID-19, *JAMA* 2020;323:1502-3 doi:10.1001/jama.2020.2783 [published Online First: April 21,].

12 Tay MZ, Poh CM, Rénia L, et al. The trinity of COVID-19: immunity, inflammation and intervention, *Nature reviews. Immunology* 2020;20:363-74 doi:10.1038/s41577-020-0311-8 [published Online First: Jun].

13 Colafrancesco S, Alessandri C, Conti F, et al. COVID-19 gone bad: A new character in the spectrum of the hyperferritinemic syndrome? *Autoimmunity Reviews* 2020;19:102573 doi:10.1016/j.autrev.2020.102573 [published Online First: July 1,].

For peer review only - http://bmjopen.bmj.com/site/about/guidelines.xhtml

14 Landi F, Gremese E, Bernabei R, et al. Post-COVID-19 global health strategies: the need for an interdisciplinary approach, *Aging clinical and experimental research* 2020;32:1613-20 doi:10.1007/s40520-020-01616-x [published Online First: Aug].

15 Forte G, Favieri F, Tambelli R, et al. COVID-19 Pandemic in the Italian Population:Validation of a Post-Traumatic Stress Disorder Questionnaire and Prevalence of PTSD Symptomatology, *International Journal of Environmental Research and Public Health* 2020 [published Online First: June 10,].

16 Jiang H, Nan J, Lv Z, et al. Psychological impacts of the COVID-19 epidemic on Chinese people: Exposure, post-traumatic stress symptom, and emotion regulation, *Asian Pacific Journal of Tropical Medicine* 2020;13:252 doi:10.4103/1995-7645.281614 [published Online First: 6/1/].

17 Nalbandian A, Sehgal K, Gupta A, et al. Post-acute COVID-19 syndrome, *Nature medicine* 2021;27:601-15 doi:10.1038/s41591-021-01283-z [published Online First: Apr].

18 Taquet M, Geddes JR, Husain M, et al. 6-month neurological and psychiatric outcomes in 236 379 survivors of COVID-19: a retrospective cohort study using electronic health records, *The Lancet. Psychiatry* 2021;8:416-27 doi:10.1016/S2215-0366(21)00084-5 [published Online First: May].

19 Michelen M, Cheng V, Manoharan L, et al. Characterising long term Covid-19: a living systematic review. 2020.

20 Ray STJ, Abdel-Mannan O, Sa M, et al. Neurological manifestations of SARS-CoV-2 infection in hospitalised children and adolescents in the UK: a prospective national cohort study, *The Lancet Child & Adolescent Health* 2021 doi:10.1016/S2352-4642(21)00193-0 [published Online First: July 15,].

21 Kanberg N, Ashton NJ, Andersson L, et al. Neurochemical evidence of astrocytic and neuronal injury commonly found in COVID-19, *Neurology* 2020;95:e1754-9 doi:10.1212/WNL.0000000000010111.

22 de Melo GD, Lazarini F, Levallois S, et al. COVID-19-related anosmia is associated with viral persistence and inflammation in human olfactory epithelium and brain infection in hamsters, *Sci Transl Med* 2021;13 doi:10.1126/scitranslmed.abf8396 [published Online First: -06-02].

23 Lee M, Perl DP, Nair G, et al. Microvascular Injury in the Brains of Patients with Covid-19, *New England Journal of Medicine* 2021;384:481-3 doi:10.1056/NEJMc2033369 [published Online First: February 4,].

24 Alvarez Bravo G, RamióTorrentà L. Encefalitis anti-NMDA-R secundaria a infección por SARS-CoV-2 Anti–NMDA receptor encephalitis secondary to SARS-CoV-2 infection, *Neurología (Barcelona, English ed.)* doi:10.1016/j.nrleng.2020.07.011.

25 Philippens, Ingrid H. C. H. M., Böszörményi KP, Wubben JA, et al. SARS-CoV-2 causes brain inflammation and induces Lewy body formation in macaques, *bioRxiv* 2021:2021.02.23.432474 doi:10.1101/2021.02.23.432474.

For peer review only - http://bmjopen.bmj.com/site/about/guidelines.xhtml

26 Douaud G, Lee S, Alfaro-Almagro F, et al. Brain imaging before and after COVID-19 in UK Biobank, *medRxiv* 2021 doi:10.1101/2021.06.11.21258690 [published Online First: -06-20].

27 Ladds E, Rushforth A, Wieringa S, et al. Developing services for long COVID: lessons from a study of wounded healers, *Clinical medicine (London, England)* 2021;21:59-65 doi:10.7861/clinmed.2020-0962 [published Online First: Jan].

28 Chase SE. Narrative Inquiry: Towards theoretical and methodological maturity. In: Denzin N, Lincoln Y, eds. The Sage Handbook of Qualitative Research. London: SAGE 2018.

29 Kamberelis G, Dimitriadis G, Welker A. Focus Group Research and/in Figured Worlds.

30 Glaser BG. The Constant Comparative Method of Qualitative Analysis, *Social Problems* 1965;12:436-45 doi:10.2307/798843.

31 Meinhardt J, Radke J, Dittmayer C, et al. Olfactory transmucosal SARS-CoV-2 invasion as a port of central nervous system entry in individuals with COVID-19, *Nature neuroscience* 2021;24:168-75 doi:10.1038/s41593-020-00758-5 [published Online First: Feb].

32 Stephan KE, Manjaly ZM, Mathys CD, et al. Allostatic Self-efficacy: A Metacognitive Theory of Dyshomeostasis-Induced Fatigue and Depression, *Front Hum Neurosci* 2016;10 doi:10.3389/fnhum.2016.00550.

33 May CR, Eton DT, Boehmer K, et al. Rethinking the patient: using Burden of Treatment Theory to understand the changing dynamics of illness, *BMC health services research* 2014;14:281 doi:10.1186/1472-6963-14-281 [published Online First: Jun 26,].

34 Bury M. The sociology of chronic illness: a review of research and prospects, *Sociology of Health & Illness* 1991;13:451-68 doi:10.1111/j.1467-9566.1991.tb00522.x.

35 Corbin J, Strauss A. Managing Chronic Illness at Home: Three Lines of Work, *Qualitative sociology* 1985;8:224-47 doi:10.1007/BF00989485.

36 Frank A. The Wounded Storyteller 1995.

37 Scambler G. Health-related stigma, *Sociology of Health & Illness* 2009;31:441-55 doi:https://doi.org/10.1111/j.1467-9566.2009.01161.x.

38 Bate P, Robert G. Experience-based design: from redesigning the system around the patient to co-designing services with the patient, *BMJ Quality & Safety* 2006;15:307-10 doi:10.1136/qshc.2005.016527 [published Online First: /10/01].

39 Schei E. Doctoring as Leadership: the power to heal, *Perspectives in Biology and Medicine* 2006;49:393-406 doi:10.1353/pbm.2006.0048.

40 Frank A. Just Listening: Narrative and Deep Illness, *Families, Systems, & Health* 1998;16:197-212 doi:10.1037/h0089849 [published Online First: September 1,].

For peer review only

41 Heath I. Following the story: continuity of care in general practice. In: Anonymous . Narrative Based Medicine: dialogue and discourse in clinical practice. Londo: BMJ Books 1998:83-92.

42 Office for National Statistics. Research report on population estimates by ethnic group and religion
. 2019.

43 Goffman E. Stigma: notes on the management of spoiled identity. Englewood Cliffs, N.J.: Prentice-Hall 1963.

44 Scambler G, Hopkins A. Being epileptic: coming to terms with stigma, *Sociology of Health & Illness* 1986;8:26-43 doi:10.1111/1467-9566.ep11346455.

45 Fitzgerald MH, Paterson KA. The hidden disability dilemma for the preservation of self, *Journal of Occupational Science* 1995;2:13-21 doi:10.1080/14427591.1995.9686392 [published Online First: April 1,].

46 Valeras A. "We don't have a box": Understanding Hidden Disability Identity Utilizing Narrative Research Methodology, *Disability Studies Quarterly* 2010;30 doi:10.18061/dsq.v30i3/4.1267 [published Online First: /08/24].

47 Kk O, Am B, C S, et al. Exploring disability from the perspective of adults living with HIV/AIDS: development of a conceptual framework. *Health Qual Life Outcomes* 2008;6:76-doi:10.1186/1477-7525-6-76 [published Online First: /10/04].

48 R C. The patient-physician relationship. Narrative medicine: a model for empathy, reflection, profession, and trust. *JAMA* 2001;286:1897-902 doi:10.1001/jama.286.15.1897 [published Online First: /10/01].

49 Al-Sarraj S, Troakes C, Hanley B, et al. The spectrum of neuropathology in COVID-19, *Neuropathology and Applied Neurobiology* 2021;47:3-16 doi:https://doi.org/10.1111/nan.12667.

50 Tay MZ, Poh CM, Rénia L, et al. The trinity of COVID-19: immunity, inflammation and intervention, *Nature reviews. Immunology* 2020;20:363-74 doi:10.1038/s41577-020-0311-8 [published Online First: Jun].

51 Paniz-Mondolfi A, Bryce C, Grimes Z, et al. PMC7264598; Central nervous system involvement by severe acute respiratory syndrome coronavirus-2 (SARS-CoV-2), *J Med Virol* 2020;92:699-702 doi:10.1002/jmv.25915 [published Online First: Jul].

52 Matschke J, Lütgehetmann M, Hagel C, et al. Neuropathology of patients with COVID-19 in Germany: a post-mortem case series, *The Lancet Neurology* 2020;19:919-29 doi:10.1016/s1474-4422(20)30308-2.

53 Netland J, Meyerholz DK, Moore S, et al. PMC2493326; Severe acute respiratory syndrome coronavirus infection causes neuronal death in the absence of encephalitis in mice transgenic for human ACE2, *J Virol* 2008;82:7264-75 doi:10.1128/JVI.00737-08 [published Online First: Aug].

For peer review only

54 Marlow LL, Faull OK, Finnegan SL, et al. PMC6686955; Breathlessness and the brain: the role of expectation, *Curr Opin Support Palliat Care* 2019;13:200-10 doi:10.1097/SPC.0000000000000441 [published Online First: Sep].

55 Yong SJ. PMC7874499; Persistent Brainstem Dysfunction in Long-COVID: A Hypothesis, *ACS Chem Neurosci* 2021;12:573-80 doi:10.1021/acschemneuro.0c00793 [published Online First: Feb 17].

56 Tarlov AR. Social determinants of health : The sociobiological translation: Routledge 2002:87-109.

57 Ferrucci R, Dini M, Groppo E, et al. PMC7917789; Long-Lasting Cognitive Abnormalities after COVID-19, *Brain Sci* 2021;11 doi:10.3390/brainsci11020235 [published Online First: Feb 13].

58 Jaywant A, Vanderlind WM, Alexopoulos GS, et al. PMC7884062; Frequency and profile of objective cognitive deficits in hospitalized patients recovering from COVID-19, *Neuropsychopharmacology* 2021 doi:10.1038/s41386-021-00978-8 [published Online First: Feb 15].

59 Sheng B, Cheng SK, Lau KK, et al. PMC7135192; The effects of disease severity, use of corticosteroids and social factors on neuropsychiatric complaints in severe acute respiratory syndrome (SARS) patients at acute and convalescent phases, *Eur Psychiatry* 2005;20:236-42 doi:10.1016/j.eurpsy.2004.06.023 [published Online First: May].

60 Hickie I, Davenport T, Wakefield D, et al. PMC1569956; Post-infective and chronic fatigue syndromes precipitated by viral and non-viral pathogens: prospective cohort study, *BMJ* 2006;333:575 doi:10.1136/bmj.38933.585764.AE [published Online First: Sep 16].

61 Cairns V, Godwin J. Post-Lyme borreliosis syndrome: a meta-analysis of reported symptoms, *Int J Epidemiol* 2005;34:1340-5 doi:10.1093/ije/dyi129 [published Online First: Dec].

62 National Institute for Health and Care Excellence. Diagnosis of CFS. 2020;2021.

63 Institute of Medicine. IOM 2015 Diagnostic Criteria | CDC. 2015;2021.

64 Carruthers BM, van de Sande M,I., De Meirleir K,L., et al. Myalgic encephalomyelitis: international consensus criteria, 2011;270:327-38.

65 Wei J, Hou J, Su B, et al. The Prevalence of Frascati-Criteria-Based HIV-Associated Neurocognitive Disorder (HAND) in HIV-Infected Adults: A Systematic Review and Meta-Analysis, *Front Neurol* 2020;11 doi:10.3389/fneur.2020.581346.

66 Yang AC, Kern F, Losada PM, et al. Dysregulation of brain and choroid plexus cell types in severe COVID-19, *Nature* 2021;595:565-71 doi:10.1038/s41586-021-03710-0 [published Online First: /07].

For peer review only

For peer review only - http://bmjopen.bmj.com/site/about/guidelines.xhtml

67 BERGER Z, ALTIERY DE JESUS V, ASSOUMOU SA, et al. Long COVID and Health Inequities: The Role of Primary Care, *The Milbank quarterly* 2021;99:519-41 doi:10.1111/1468-0009.12505 [published Online First: Jun].

68 Greenhalgh T, Knight M, A'Court C, et al. Management of post-acute covid-19 in primary care, *BMJ* 2020;370:m3026 doi:10.1136/bmj.m3026.

69 Kingstone T, Taylor AK, O'Donnell CA, et al. Finding the 'right' GP: a qualitative study of the experiences of people with long-COVID, *BJGP Open* 2020;4:bjgpopen20X101143 doi:10.3399/bjgpopen20X101143.

For peer review only

BMJ Open

“I can’t cope with multiple inputs”: Qualitative study of the lived experience of ‘brain fog’ after Covid-19

Journal:	*BMJ Open*
Manuscript ID	bmjopen-2021-056366.R1
Article Type:	Original research
Date Submitted by the Author:	03-Dec-2021

Complete List of Authors:	Callan, Caitriona ; University of Oxford Nuffield Department of Primary Care Health Sciences<br>ladds, emma; University of Oxford Nuffield Department of Primary Care Health Sciences,<br>Husain, Laiba; University of Oxford, Nuffield Department of Primary Care Health Sciences<br>Pattinson, Kyle; University of Oxford, Nuffield Department of Clinical Neurosciences<br>Greenhalgh, Trisha; University of Oxford, Nuffield Department of Primary Care Health Sciences

**Primary Subject Heading**:	General practice / Family practice
Secondary Subject Heading:	Rehabilitation medicine
Keywords:	COVID-19, INFECTIOUS DISEASES, OCCUPATIONAL & INDUSTRIAL MEDICINE, QUALITATIVE RESEARCH

SCHOLARONE™
Manuscripts

I, the Submitting Author has the right to grant and does grant on behalf of all authors of the Work (as defined in the below author licence), an exclusive licence and/or a non-exclusive licence for contributions from authors who are: i) UK Crown employees; ii) where BMJ has agreed a CC-BY licence shall apply, and/or iii) in accordance with the terms applicable for US Federal Government officers or employees acting as part of their official duties; on a worldwide, perpetual, irrevocable, royalty-free basis to BMJ Publishing Group Ltd ("BMJ") its licensees and where the relevant Journal is co-owned by BMJ to the co-owners of the Journal, to publish the Work in this journal and any other BMJ products and to exploit all rights, as set out in our licence.

The Submitting Author accepts and understands that any supply made under these terms is made by BMJ to the Submitting Author unless you are acting as an employee on behalf of your employer or a postgraduate student of an affiliated institution which is paying any applicable article publishing charge ("APC") for Open Access articles. Where the Submitting Author wishes to make the Work available on an Open Access basis (and intends to pay the relevant APC), the terms of reuse of such Open Access shall be governed by a Creative Commons licence – details of these licences and which Creative Commons licence will apply to this Work are set out in our licence referred to above.

Other than as permitted in any relevant BMJ Author's Self Archiving Policies, I confirm this Work has not been accepted for publication elsewhere, is not being considered for publication elsewhere and does not duplicate material already published. I confirm all authors consent to publication of this Work and authorise the granting of this licence.

For peer review only

Original article resubmitted to BMJ Open November 2021

"I can't cope with multiple inputs": Qualitative study of the lived experience of 'brain fog' after Covid-19

For peer review only

Caitriona Callan MBBChir MA(Cantab)[1], academic clinical fellow

Emma Ladds MBBCh MPH MRCGP[1,] academic clinical fellow

Laiba Husain BA MPH[1], research assistant

Kyle Pattinson BM DPhil FRCA[2], clinical professor

Trisha Greenhalgh MBBCh MD FRCGP FRCP FFPH MBA[1], clinical professor

[1] Nuffield Department of Primary Care Health Sciences, University of Oxford

[2] Nuffield Department of Clinical Neurosciences, University of Oxford

Corresponding author:

E Ladds: e.ladds@nhs.net

Nuffield Department of Primary Care Health Sciences

University of Oxford, Oxford OX2 6GG

UK

For peer review only - http://bmjopen.bmj.com/site/about/guidelines.xhtml

Key words: Ongoing symptomatic Covid-19, Post-Covid-19 syndrome, long Covid, qualitative study, neurocognitive

For peer review only

For peer review only - http://bmjopen.bmj.com/site/about/guidelines.xhtml

Abstract

Objective

To explore the lived experience of 'brain fog'—the wide variety of neurocognitive symptoms that can follow Covid-19.

Design and setting

UK-wide longitudinal qualitative study comprising online focus groups with email follow-up.

Method

50 participants were recruited from a previous qualitative study of the lived experience of long Covid (n = 23) and online support groups for people with persistent neurocognitive symptoms following Covid-19 (n = 27). In remotely-held focus groups, participants were invited to describe their neurocognitive symptoms and comment on others' accounts. Individuals were followed up by email 4-6 months later. Data were audiotaped, transcribed, anonymised and coded in NVIVO. They were analysed by an interdisciplinary team with expertise in general practice, clinical neuroscience, the sociology of chronic illness and service delivery, and checked by people with lived experience of brain fog.

Results

Of the 50 participants, 42 were female and 32 White British. Most had never been hospitalised for Covid-19. Qualitative analysis revealed the following themes: mixed views on the appropriateness of the term 'brain fog'; rich descriptions of the experience of neurocognitive symptoms (especially executive function, attention, memory and language),

For peer review only - http://bmjopen.bmj.com/site/about/guidelines.xhtml
For peer review only

accounts of how the illness fluctuated—and progressed over time; the profound psychosocial impact of the condition on relationships, personal and professional identity; self-perceptions of guilt, shame and stigma; strategies used for self-management; challenges accessing and navigating the healthcare system; and participants' search for physical mechanisms to explain their symptoms.

Conclusion

These qualitative findings complement research into the epidemiology and mechanisms of neurocognitive symptoms after Covid-19. Services for such patients should include: an ongoing therapeutic relationship with a clinician who engages with their experience of neurocognitive symptoms in its personal, social and occupational context as well as specialist services that include provision for neurocognitive symptoms, are accessible, easily navigable, comprehensive, and interdisciplinary.

For peer review only

Summary

Strengths and Limitations of Study

- To our knowledge, this is the largest and most in-depth qualitative study of the lived experience of brain fog in survivors of Covid-19.
- The research team was interdisciplinary and interprofessional, and included consultation with two patient experts by experience suffering from ongoing, improving brain fog, who helped with data interpretation and peer review.
- Oversampling from men and non-white ethnic groups allowed partial correction of an initially skewed sample.
- The sample was drawn entirely from the UK
- Residual skews in the samples, particularly regarding minority ethnic groups and occupational classes and the digitally excluded, limited our ability to capture the full range of experiences

Funding statement

This research is funded from the following sources: National Institute for Health Research (BRC-1215-20008), ESRC (ES/V010069/1), and Wellcome Trust (WT104830MA). Funders had no say in the planning and execution of the study or writing up of the paper. KTSP is supported by the National Institute for Health Research Biomedical Research Centre based at Oxford University Hospitals NHS Foundation Trust and the University of Oxford.

Competing Interests Statement

EL and TG provided evidence on long Covid for House of Lords Select Committee

For peer review only - http://bmjopen.bmj.com/site/about/guidelines.xhtml

TG was on the oversight group for the National Institute for Health and Clinical Excellence guideline on managing the long-term effects of Covid-19, and at the time of writing is on the UK's National Long Covid Task Force.

KP and CC have no competing interests to declare.

For peer review only

For peer review only - http://bmjopen.bmj.com/site/about/guidelines.xhtml

Background

It is now well-established that symptoms can occur beyond acute COVID-19. Results from a UK sample suggest 1 in 10 people self-report ongoing, otherwise unexplained symptoms 12 weeks after infection [1]. Over half experience a reduced functionality for everyday activities and many remain unable to work weeks after infection [2]. The growing frequency of chronic and/or disabling illness related to COVID-19 has rendered their health needs, and associated clinical and occupational guidelines, policy priorities [3-5].

Long Covid, a "patient-made" term [6], embraces the formally-defined ongoing symptomatic Covid-19 syndrome (symptoms persisting between 4-12 weeks) and post-Covid-19 syndrome (symptoms beyond 12 weeks)[5]. In this paper we use 'long Covid' to refer to the lived patient experience and 'post-Covid-19 syndrome' to refer to the medically diagnosed condition. It is highly heterogenous with sufferers reporting a range of fluctuating symptoms, amongst which fatigue, breathlessness, chest pain, post-exertional malaise, autonomic nervous system disruption, and cognitive dysfunction [4, 7-9] are common. The pathophysiology remains unclear, however persistent viraemia [10], relapse or reinfection [11] inflammatory and immune reactions [12, 13], deconditioning [14] and psychological factors [15, 16] have been proposed as contributors. It is likely causative pathways are multifactorial [17].

Analysis of a quarter of a million Covid-19 survivors' health records revealed widespread neurological and psychiatric presentations with around a third persistently affected over the following 6 months [18]. Around one-quarter experienced disturbed mood, and a fraction developed serious problems such as psychosis. Other neurological problems have included

For peer review only

cerebrovascular events, encephalitis, dementia, and disorders of peripheral nerves, nerve roots or plexuses [18]. Surveys and focus groups of online, non-hospitalised long Covid patients have identified subjective and/or objectively measured impairments in attentional processing, short-term memory and executive function, alongside a befuddled state termed ’brain fog’ by many patients [4, 7, 9, 19]. A few studies have explored correlations between subjective cognitive dysfunction and neuropsychological testing deficits with mixed findings [20-22].

In this paper we use patients’ own descriptions of their symptoms (using their term ‘brain fog’) and, when appropriate, the US National Cancer Institute definition of ‘neurocognitive symptoms’ to describe subjective problems “to do with the ability to think and reason, [including] the ability to concentrate, remember things, process information, learn, speak, and understand”[23]. Possible proposed biological factors include direct neuroinvasion [24], viral persistence and chronic inflammation [25], neuronal injury or toxicity and glial activation [24, 26], microvascular injury [27], activation of autoimmune mechanisms [28], and Lewy body production [29], whilst imaging demonstrates loss of grey matter in Covid patients in key brain regions [30].

The functional impact of such neurocognitive symptoms is often profound, affecting individuals’ abilities to work and perform daily activities [4, 9], increasing healthcare contacts [31], impeding decision making, communication and social relationships. UK clinical guidelines suggest that clinical psychology and psychiatry specialists should be part of the multidisciplinary team conducting post-Covid rehabilitation [5] but these are contested and inconsistently implemented. Developments in treatment approaches, service pathways,

For peer review only - http://bmjopen.bmj.com/site/about/guidelines.xhtml

and occupational supports require better understanding of underlying causal and contributory factors as well as the lived experience of sufferers. 'Brain fog' has been highlighted in previous research as a particularly impactful aspect of long Covid which sufferers are keen to have further explored [4, 32, 33].

In this study, we sought to answer three key questions: a) what neurocognitive symptoms are experienced by adults with long Covid?; b) what is the impact of these symptoms?; and c) how do individuals deal with them? We also sought to explore whether our understanding of cognitive processes/perceptions and the Covid-19 virus could inform potential causative explanations.

Methods

Study design and governance

This study extended a previous qualitative study of 114 people with self-defined long Covid [4, 32]. Ethical approval was granted from the East Midlands – Leicester Central Research Ethics Committee (IRAS Project ID: 283196; REC ref 20/EM0128) on 4th May 2020 and subsequent amendments. Original recruitment took place between May and September 2020 from support groups on Facebook, a social media call (Twitter), and snowballing. To correct skew, men and minority ethnic groups were oversampled. In October 2020, partly prompted by participants' desire to further explore brain fog, the original sample were emailed for focus group volunteers - 23 agreed. 27 additional participants were then recruited from an online support group dedicated to long Covid's neurocognitive effects. The dataset for this study thus consisted of data from the original study and focus groups from the new sample of

For peer review only - http://bmjopen.bmj.com/site/about/guidelines.xhtml

50. In line with ethics committee recommendations and infection control measures email or verbal consent was obtained [4].

Five focus groups of 60-90 minute duration were held via Zoom in October and November 2020 with 10 to 14 participants. Each group had two facilitators (EL and LH - female researchers experienced in qualitative research with qualifications in general practice and public health) who also took contemporaneous notes. Participants were invited to tell the story of their neurocognitive symptoms, with conversational prompts to maintain the narrative and elicit the impact on an individual's life and any interaction between neurocognitive and other perceptually 'physical' symptoms [34]. We encouraged the sharing of stories to identify issues important to the patient, emotional touchpoints in their illness journeys, and promote interaction between participants [35].

Data management and analysis

Focus groups were videotaped with consent, transcribed in full, de-identified and entered onto NVIVO software version 12 alongside contemporaneous notes. Additional material from the original dataset was included. Sections of text were initially coded by CC (a female researcher qualified in psychology and medicine and training in qualitative methodology) into 6 categories: naming the phenomenon; lived experience of symptoms; interaction of neurocognitive and other symptoms; impact of symptoms; self-management; and experiences navigating healthcare services. These were informed by, but not limited to, the theoretical framework discussed below.

For peer review only

For peer review only - http://bmjopen.bmj.com/site/about/guidelines.xhtml

An interim synthesis was produced from early transcripts and progressively refined using the constant comparative method by CC and EL [36]. Finally, to add descriptive depth, clarify discrepancies or ambiguities within the data and track progression of symptoms, we sent each participant a follow-up email 4-6 months later (10-12 months after their acute illness). We asked how their symptoms were progressing and to describe their current neurocognitive symptoms. 20 participants responded and this data was integrated into, and refined, our final interpretation. While saturation did not determine sample size, thematic saturation was reached [37].

Theoretical framework

Our analysis was informed by three theoretical lenses. First, we considered the symptom burden of long COVID from a neuroscience perspective. For many, long COVID symptoms are poorly explained by objective medical tests. Although this may relate to undiagnosed peripheral pathophysiology, there is an increasing appreciation that unexplained symptoms also relate to the brain's perceptual processes [38, 39]. The brain has no direct access to the body or outside world and must make sense of noisy incoming sensory signals. Current theories propose signals are deciphered by referring to an internally held model of perception [38, 39]. This can be influenced by multiple factors including mood, previous experiences and conscious or unconscious beliefs. Thus symptoms can be generated, exacerbated or perpetuated independently of a cause 'in the body' [38-40]. In the case of COVID-19, SARS-COV-2 is neuroinvasive, and thus additionally may directly disrupt these perceptual processes [38].

For peer review only - http://bmjopen.bmj.com/site/about/guidelines.xhtml

Second, sociological theories of chronic illness, including May's burden of illness theory [41], biographical perspectives on chronic illness [42-44]; and the sociological notion of stigma [45]. Third, emotional touchpoints of powerful feelings such as anger, fear, or hope [46] – particularly in participants' experiences of healthcare, which may be interpreted using theories of good professional practice [47], the therapeutic relationship [48] and continuity of care [49].

Patient involvement statement

The study was planned, undertaken, analysed, and written in collaboration with participants suffering from long Covid. All were invited to a webinar presentation sharing key findings and quotes, provided with a recording and copy of the presentation, and invited to correct errors or misinterpretations, which largely reflected a desire to ensure the severity of their symptoms and their impacts were appropriately represented. Although the recovery status of all participants is unknown, 13 of the 20 follow-up respondents had ongoing but improving brain fog 10-12 months after initial infection. Furthermore, two clinically qualified people still suffering from long Covid reviewed a near-final draft of this paper, which was modified in response.

Results

Description of dataset

Details of participants are shown in Table 1. Despite our efforts to balance for gender and ethnicity, the final sample was skewed to 42 of 50 (84%) female and 36 (72%) White. By comparison, long Covid support groups are up to 86% female [9] and the UK population is

For peer review only - http://bmjopen.bmj.com/site/about/guidelines.xhtml

80-85% White British [50]. The 5 focus groups, chat transcripts, follow-up email communications and participant webinar discussion produced over 1000 pages of transcripts and notes. The six emergent coding themes are discussed in more detail below with illustrative quotes in Table 2 and definitions of neurocognitive processes/functions in box 1.

1. Naming the phenomenon
2. Neurocognitive symptoms and their natural history
3. Neurocognitive symptoms in the context of other long Covid symptoms
4. Psychosocial impact: guilt, shame and stigma
5. Hypothesising mechanisms to inform self-management
6. Navigating healthcare

Naming the phenomenon

Participants varied in their attitudes towards the patient-made term 'brain fog' [9]. Some found it useful as an accessible and well-known shorthand to disclose their wide-ranging cognitive difficulties to others, but others felt the term lacked specificity or did not convey the severity of their symptoms (Quote 1). Alternative terms preferred by some participants included 'clinical or profound brain dysfunction', 'neurocognitive fatigue' or 'brain impairment', although all participants used the term 'brain fog' in group discussions.

Neurocognitive symptoms and their natural history

This study focussed on patients' lived experiences with no objective examination. However their descriptions often related to specific domains of cognitive function—particularly executive function, attention, memory and language, with most describing difficulties across all of these domains. Participants described problems with planning, decision-making,

For peer review only - http://bmjopen.bmj.com/site/about/guidelines.xhtml
For peer review only

flexibility and working memory which concorded with executive function cognitive processes (Quote 2), whilst impairments in complex attention included difficulties with selective, sustained attention, divided attention, and processing speed (Quote 3), and long-term memory impairments were experienced with free recall, cued recall, and procedural memory (Quote 4). Language deficits varied between individuals, including difficulties with word-finding and fluency, syntax, reading comprehension and writing (Quote 5).

The longitudinal email follow-up allowed us to explore some aspects of the condition's natural course. Most respondents reported emergence of neurocognitive symptoms 1-4 months after their initial illness, and 13/20 felt they had improving brain fog at time of follow-up. Neurocognitive symptoms tended to fluctuate diurnally and over weeks to months, typically, but not invariably, showing gradual long-term improvement (Quote 6). The tiring and unpredictable nature of the symptoms were destabilising and debilitating and were reported similarly amongst all participants.

Neurocognitive symptoms in the context of other long Covid symptoms

Participants described having distinct experiences of 'neurocognitive' compared to 'physical' symptoms. The latter were generally presented as somatic manifestations, often familiar from other conditions, such as physical fatigue, tachycardia, or breathlessness. Despite this distinction, there was a recognition that both 'physical' and 'neurocognitive' symptoms were often associated or interacting. Many highlighted the fatiguability of their neurocognitive or physical symptoms from either mental or physical effort (Quote 7).

For peer review only

For peer review only - http://bmjopen.bmj.com/site/about/guidelines.xhtml

Psychosocial impact: guilt, shame and stigma

Participants described profound psychological, occupational and social impacts. Several had been unable to return to work at their previous level or at all. Participants who had returned to work described adopting reduced hours or adapted roles, often associated with anxiety about potential risks associated with mistakes in cognitively demanding or high-responsibility roles (Quote 3), self-doubt about their abilities, loss of self-worth, and altered identities (Quote 8).

Participants reported how their symptoms induced strong emotional responses in themselves and others. Guilt and shame were particularly evident, often relating to difficulties in returning to work, their previous level of function, or a lack of understanding from others (Quotes 9 & 10). Particularly troubling were physically invisible deficits, such as difficulties with language or memory. Participants also described instances of conflict arising from their impaired cognition (Quote 12).

Hypotheses to inform self-management

Participants frequently attempted to make sense of their symptoms and communicate the severity and legitimacy of their suffering through analogous referral to disorders with accepted mechanisms such as stroke, concussion or dementia (Quotes 14 & 16). Although of those who had been ‘investigated’ many were ‘normal’, participants were keen to hypothesise about biological explanations for their symptoms with some also mentioning psychological contributors to their experience. Some reported various self-management strategies based on hypothetical mechanisms such as dietary adaptations (Quote 6), food supplements or complementary therapies, which were met with variable success.

For peer review only

For peer review only - http://bmjopen.bmj.com/site/about/guidelines.xhtml

Many had developed coping strategies to deal with their neurocognitive symptoms, centred around self-expectation management and rest prioritisation, resulting in self-negotiations and activity trade-offs, which were frustrating and psychologically draining (Quote 11). Moreover, conveying their reduced and variable cognitive function to family, friends or colleagues was a significant challenge and some developed innovative communication strategies (Quote 12).

For peer review only

Navigating the healthcare system

Participants had varying experiences of healthcare systems, with impaired memory and word-finding issues adding to the challenge of communication and self-advocacy (Quote 13). Moreover, articulating the specifics of the 'brain fog' experience to healthcare professionals was a particular issue and frustration, anger and hopelessness were commonly experienced when the impact of neurocognitive symptoms was 'downplayed', dismissed as being all 'in your head' or secondary to depression or anxiety, or deprioritised relative to other Covid sequelae. Some participants perceived being middle-age and female as contributing to their not being taken seriously by healthcare professionals.

Conversely, some participants described huge relief and validation at feeling believed and acknowledged (Quote 14), particularly in the context of continuity, wise counselling, and healthcare professionals bearing witness within therapeutic relationships (Quote 15). Several participants had undergone brain imaging or neuropsychological testing, which were overwhelmingly normal and thus often enabled participants to focus on self-management, frequently supported by allied health professionals including occupational therapists and physiotherapists. None reported having seen a psychologist or psychiatrist in any context.

For peer review only - http://bmjopen.bmj.com/site/about/guidelines.xhtml

Discussion

Summary of key findings

This qualitative study of 50 UK participants suffering from neurocognitive symptoms following Covid-19 has revealed several important findings. Subjective impairments in executive function, attention, memory, and language were common, often emerging weeks to months after the acute illness and in most cases following a relapsing-remitting course that gradually improved over months. Prominent fatiguability and interaction between perceptually cognitive or physical symptoms combined with the impact on professional and personal activities, functional ability and identities to produce a destabilising, debilitating, frustrating, stigmatising and frightening situation. Variably successful approaches to mitigate the effect of brain fog included activity trade-offs and communication strategies and the experience of illness was greatly compounded by the challenges in navigating the healthcare system when subjectively cognitively impaired.

Comparison with theoretical literature

Some accounts of the condition fitted Frank's definition of the 'chaos narrative', where the illness experience is unresolved by restitution of the former healthy self thus remains confusing and lacking in meaning [44]. The profound impact of symptoms on individuals' independence, self-efficacy, and self-trust resonated with descriptions of spoiled identity and the disrupted sense of purpose and self that can accompany chronic illness [51], whilst others aligned with theoretical accounts of shame and blame in other partly-invisible conditions such as epilepsy [52].

For peer review only - http://bmjopen.bmj.com/site/about/guidelines.xhtml

Participants' concerns also reflected the phenomenon of 'hidden disability', whereby individuals must undergo a contextual negotiation about when to 'pass' as able-bodied, and when to self-identify as having a disability. In so doing they must weigh up conflicting drivers of self-identity and preservation of self, impression management, stigma, and legitimization of or possible value judgements based on illness-related behaviour [53, 54]. Moreover, the relapsing-remitting time course of brain fog symptoms also align with 'episodic disability', as described by those with HIV, to describe unpredictable periods of wellness and illness [55], which adds an additional element of uncertainty.

Such requirements emphasize the extensive work people with long Covid must undertake to manage their condition and navigate services, according with theories of illness burden [41], which, until recently has been compounded by the lack of clear care pathways [4]. Positive experiences of care described dimensions of good professional practice: active listening and bearing witness [48, 56]; wise counsel [47] and continuity of the therapeutic relationship [49] that alleviate patients' illness burden and help begin to construct a healing narrative.

Lack of understanding about the cause of neurocognitive symptoms was a frequent frustration for participants. Ongoing research has hypothesized neuronal damage occurs secondary to direct viral neurotoxicity [57] or associated neuroinflammation that generates a multisystem dysfunction resulting from a loss of central control and generalized peripheral inflammatory response [58]. Such suggestions are supported by pathological evidence of SARS-CoV-2 neurotropism [59] and neuroinflammation [60] combined with animal models of SARS-CoV-2 infection leading to neuroinflammation, intracellular Lewy body formation, or neuronal loss [29, 61]. It has been hypothesized that such processes impacting on vulnerable brain

For peer review only - http://bmjopen.bmj.com/site/about/guidelines.xhtml

regions could correlate with neurocognitive symptoms in ongoing Covid-19 or post-Covid-19 syndrome: dysfunction of the brain stem, which is involved in regulation of both respiration and arousal – and thus potentially 'brain fog' – could account for some of the attentional deficits and disproportionate breathlessness seen in post-Covid-19 syndrome [38, 62]. All of these theories need further research and correlation with the lived experiences reported in this study.

Finally, our findings illustrate that whatever the explanation for ongoing neurocognitive symptoms, the resultant impacts result from – and contribute to – a wider interplay of psychological, physical and social factors. The clear disruption to an individual's professional self, interpersonal relationships, and overall sense of identity, combined with hidden and episodic disabilities impair sufferers' abilities to achieve Tarlov's anticipated state of 'health', described as 'the capacity, relative to potential and aspirations, for living fully in the social environment'[63]. Given that post-Covid-19 syndrome seems more prevalent amongst those of working age, in education [64], and particularly exposed 'key worker' groups [64], the potential impact on society is significant. Therefore, whilst further work must deepen and exploit our mechanistic understanding, commissioners and providers of post-Covid 19 services, individual clinicians, and employers must remain cognizant of the disruption to these broader components of health and consider how they may be mitigated to aid recovery.

Strengths and limitations of the study

To our knowledge, to date this is the largest, most in-depth qualitative study of neurocognitive symptoms of post-Covid-19 syndrome. The research team included clinicians and social scientists. Our participants spanned a range of ages, ethnicities, social

For peer review only - http://bmjopen.bmj.com/site/about/guidelines.xhtml

backgrounds, and illness experiences – including the majority who were never hospitalised and a range of recovery states. Importantly recovery state did not seem to affect individual perceptions or recollections of brain fog, which were described consistently. The majority of our participants were infected during the initial pandemic wave, thus email follow-up almost 12 months post-infection gives a meaningful insight into the condition's natural history. We oversampled men and people from non-White groups to partially correct an initially skewed sample. The use of multiple linked sociological theories allowed rich theorisation of the lived experience of the illness, supported by input from experts by experience.

The study does have limitations. The entirely UK-based sample included a high proportion of people recruited from a support group for those with neurocognitive symptoms of long Covid, thus likely to be more severely affected and potentially suffering from higher levels of distress [65]. Moreover, our sample did not extend to all demographic subgroups so we may not have fully captured the perspectives of some minority ethnic groups, occupational classes, or those less digitally connected. In the time since the first wave, knowledge and treatment of acute Covid-19 and post-Covid-19 syndrome have altered substantially with medical research, patient advocacy, and (geographically variable) service development, which may influence the experience of long Covid for people infected at later time points.

Comparison with previous empirical studies

Our findings of persistent, debilitating neurocognitive symptoms in people living with long Covid align with several retrospective cohort studies [18] and online patient surveys [7, 9, 66,

For peer review only - http://bmjopen.bmj.com/site/about/guidelines.xhtml

67]. Our study adds further context to explore the functional and psychosocial impact of such symptoms and mitigating efforts by patients.

Comparisons have been made between post-Covid-19 syndrome and other post-infective syndromes of neurocognitive dysfunction. Infection with SARS-CoV-1 [68], Epstein-Barr Virus, Coxiella burnetii, Ross River virus [69], and Borrelia burgdoferi [70] can be associated with similar impairments to concentration and memory, typically correlated with persistent fatigue, although the causality of this association has been disputed. This study was not designed to compare the symptomatology of neurocognitive symptoms in people with long Covid to other conditions. However, the challenge of unpicking the aetiology of brain fog is illustrated by the example of chronic fatigue syndrome/myalgic encephalomyelitis (CFS/ME), where persistent difficulties with executive function, short-term memory, attention and word-finding are incorporated in the diagnostic criteria of both the UK National Institutes for Clinical Excellence [71], US Centers for Disease Control and Prevention [72], and International Consensus Group [73], but where the cause(es) of these symptoms remain unclear [74].

For peer review only

Examples such as HIV-associated neurocognitive dysfunction, which afflicts over 40% of people with chronic HIV infection [75], impairing learning, memory, attention, and executive function, suggest possible overlap across multiple chronic viral infections. A recent study in Nature illustrates how such higher order disruptions may be mediated on a molecular level through viral-associated perturbations in general cellular functions such as cortical excitatory synaptic signalling, choroid plexus disruption enabling peripheral T cell infiltration, and

For peer review only - http://bmjopen.bmj.com/site/about/guidelines.xhtml

promotion of pathological microglial and astrocyte subpopulations [76]. All of these mechanisms – and others – will require further elucidation.

Both the partially hidden nature of neurocognitive symptoms, and the extensive work required to manage these and navigate services may contribute to the ongoing dispute about how common persistent symptoms are following Covid-19 infection. Data from the Office for National Statistics have demonstrated that self-reported long Covid was greatest in people aged 35-69 years, women, people living in the most deprived areas, those in health and social care occupations, and those with another activity-limiting health condition or disability [64]. As for the acute infection, long-term sequelae of Covid-19 infection are strongly impacted by socioeconomic determinants such as poverty and structural inequalities such as racism and discrimination [77], which may affect health beliefs, health-seeking behaviours, or the response of health services. Whilst not directly reported by participants in this study, further work to explore the impact of such determinants on long Covid epidemiology and interactions with health services will be crucial to mitigate the impact of associated disability.

Conclusion: implications for services and further research

In dealing with Covid-19 it is crucial that health policy begins to shift from an acute disaster response to chronic crisis management. This study brought neuroscientists and qualitative researchers together to align the subjective illness experience with the perception of neurocognitive symptoms and proposed causal and contributory hypotheses. The profoundly disabling, persistent impacts of post-Covid-19 syndrome in a minority of people adds weight to arguments that prevention of Covid-19 reduces not only mortality but also the long-term burden of disease on patients, the health service, and the wider economy. Moreover, a better

For peer review only - http://bmjopen.bmj.com/site/about/guidelines.xhtml

understanding of the pathophysiological mechanisms and further exploration of the best approaches to support cognitive, psychological, and occupational restoration, is crucial to aid those already affected.

The strong positive and negative emotional touchpoints [46] described by individuals when their accounts are—respectively— perceived as acknowledged or dismissed underscores the importance of the clinical relationship in which the patient is listened to, their experience believed, and supported — particularly in primary care, which is likely to be the patient's first point of contact [78, 79]. Furthermore, the varied nature of the severe impacts of neurocognitive symptoms identified in this study highlight the importance of ensuring that specialist services are accessible, easily navigable, comprehensive, and interdisciplinary—for example incorporating (where necessary) assessment and rehabilitation from clinical psychologists, cognitive neurologists, and occupational therapists [5]. Our findings affirm those of a previous study to co-design quality indicators for post-Covid 19 syndrome services, which emphasised the importance of continuity, clinical responsibility, multidisciplinary input, patient involvement, and use of evidence-based guidelines [4].

For peer review only - http://bmjopen.bmj.com/site/about/guidelines.xhtml

Acknowledgements: We thank the 50 participants for their interest and contributions, and two experts by experience for helpful comments on a draft of the paper: Sharon Taylor, child psychiatrist and honorary senior lecturer at the Central and North-West London NHS Foundation Trust and Imperial College School of Medicine and Clare Rayner, Independent Occupational Physician, Manchester. Alex Rushforth and Sietse Wieringa undertook interviews for the original study of long Covid.

For peer review only

Contributors and sources: EL and TG conceptualised and designed the study. EL, LH, CC, and KP conducted focus groups. EL and CC led data analysis, with input from LH, TG and KP and produced a first draft of the results section. EL and CC wrote the first draft of the paper which was refined by all authors. LH provided research assistant support and conducted some interviews. ST and CR provided expertise by experience and knowledge of patient-led research. CC presented findings to long Covid patient participants with assistance from EL and TG. All authors contributed to refinement of the paper provided additional references. EL is corresponding author and guarantor. EL affirms that the manuscript is an honest, accurate, and transparent account of the study being reported; that no important aspects of the study have been omitted; and that any discrepancies from the study as planned (and, if relevant, registered) have been explained.

Copyright: The Corresponding Author has the right to grant on behalf of all authors and does grant on behalf of all authors, a worldwide licence to the Publishers and its licensees in perpetuity, in all forms, formats and media (whether known now or created in the future), to i) publish, reproduce, distribute, display and store the Contribution, ii) translate the Contribution into other languages, create adaptations, reprints, include within collections and

create summaries, extracts and/or, abstracts of the Contribution, iii) create any other derivative work(s) based on the Contribution, iv) to exploit all subsidiary rights in the Contribution, v) the inclusion of electronic links from the Contribution to third party material where-ever it may be located; and vi) licence any third party to do any or all of the above.

Data Sharing Statement: Deidentified participant focus group data may be available from the corresponding author, using the correspondence contact details. This is subject to the correct ethical approvals and data sharing approvals and data governance structures being in place.

For peer review only

Table 1: Participant characteristics

	Participants recruited from previous long Covid study	Participants recruited from neuro Covid support groups	Total Brain Fog Focus Group Participants	Responders to email follow-up post-focus groups
	23	27	50	20
Gender				
• Female	15	26	42	17
• Male	8	1	8	3
Age				
• Median	48	36	43	43
• Range	31-74	29-68	29-74	31-74
Ethnicity				
• White British	16	14	30	11
• White other	3	3	6	1
• Black	1	1	2	0
• Asian	3	2	5	1
• Mixed	0	0	0	0
• Non-response	0	7	7	7

Occupation<br>• Healthcare professional<br>• Non-healthcare professional<br>• Non-response	8<br>13<br>2	8<br>11<br>8	16<br>24<br>10	5<br>9<br>6
Hospitalised at any point due to Covid-19<br>• Yes<br>• No<br>• Non-response	0<br>9<br>14	4<br>8<br>15	4<br>17<br>29	4<br>16

Box 1. Definitions

Planning: the mental process allowing individuals to choose necessary actions to reach a goal, ascertain the required order, assign tasks to cognitive resources, and establish a plan of action.

Decision making: the cognitive process resulting in the selection of a belief or a course of action from multiple possible alternative options,

Flexibility: the mental ability to adjust activity and content of the cognitive system i.e. enabling a switch between different task rules and corresponding behavioural responses, maintaining multiple concepts simultaneously and shifting internal attention between them.

Complex attention: a person's ability to maintain information in their mind for a short time and to manipulate that information eg: to perform mental arithmetic calculations.

Selective sustained attention: the ability to focus on an activity or stimulus over a long period of time even if there are other distracting stimuli present.

Divided attention: the ability to attend to multiple different stimuli at the same time, thus responding to more than one demand from the surroundings i.e. enabling multi-tasking.

Processing speed: the time it takes a person to do a mental task i.e. the at which a person can understand and react to the information they receive from sensory inputs and generate a reaction.

Working memory: a cognitive system with a limited capacity, capable of temporarily holding information to enable reasoning and guiding decision-making and behaviour.

For peer review only

Procedural memory: a type of implicit memory that aids the performance of particular types of tasks without conscious awareness of previous experiences eg: stored motor programmes of particular well-rehearsed actions.

Autobiographical memory: a memory system formed from episodes recollected from an individual's life that combines episodic (personal experiences and specific objects, people and events experienced at particular time and place) and semantic (general knowledge and facts about the world) memory.

Free recall: a common memory task requiring individuals to recall any items from a previously memorized list either immediately or following a delay.

Cued recall: As above, individuals are required to recall items from a previously memorized list but may be given cues (often semantic) to encourage this.

Table 2: Participant Quotes

Identifier	Source	Quote
1	Participant 10, Focus Group (FG) 4	"Does anyone ever refer to it as neurocognitive fatigue? In a way I don't like brain fog as it's too vague, too loose of a term, so want something more technical. Though I don't think neurocognitive fatigue encompass the word finding difficulties, so it's not ideal either"
2	Participant 7, FG1	"One of the things I've realised is how many things I do in my normal day - I'm not talking about work, just in a normal day - that are cognitive that I [didn't previously] think of as being cognitive. So a supermarket, the amount of sensory information, and just staring at a row of things looking for the food that you want, remembering where things are in the aisles and planning your trip so that you don't have to walk backwards and

For peer review only - http://bmjopen.bmj.com/site/about/guidelines.xhtml

		forwards around the shop, that surprised me. […] Not just can I walk around the supermarket, it's planning, it's getting there, it's choosing stuff, all of that is actually really difficult."
3	Participant 5, FG1	"I can't cope with multiple inputs, like if I'm trying to reply to a message on my phone and one of my boys starts speaking to me or there's something else happening as well that just really fries my brain. I mean I used to be the kind of person that, like all women, multi-tasking was a superpower. I was able to, do lots and lots of things, you know I'm [a doctor]; I would have one patient I'd be hearing lots about another patient coming I'd be remembering I'd be doing something else I'd be juggling lots and lots of things and now I can't keep multiple plates spinning I absolutely can't. I've got to focus on just one thing or I make massive mistakes and it's like I forget my intentions all the time."
4	Participant 10, FG3	"I can ask somebody a question and then I'll ask the exact same question two minutes after and not remember I've asked them, I can't remember significant things that have happened in the past either"
5	Participant 8, FG2	"[It's difficult] to comprehend and take in written information and read it. I had a form sent to me at work and I just felt, 'I can't do this at the moment' and put it to one side and hoped to come back to it because it's just been too difficult"
6	Participant 3, FG5, in email response to follow-up	"I'm probably about 90% better. I'm struggling to put in full days at work and still need a great deal of rest and sleep. My brain fog is greatly improved, although I'm making mistakes at work and

For peer review only - http://bmjopen.bmj.com/site/about/guidelines.xhtml

For peer review only

		have been forgetful and sometimes confused with large amounts of new information. I feel like my head is clear now. When you did the group interview I felt like I was drugged up all of the time. Now it's far and few days between that I feel that way. I think the brain fog lasted around eight months. I strongly believe that my improvements are diet related and have been following a low histamine diet since October"
7	Participant 2, FG1	"Sometimes I feel as though if I exert myself like cognitively then my Long Covid symptoms sort of exacerbate like shortness of breath, chest tightness. But like earlier on I think that it was the other way round […] it seemed to be that if I exert myself physically-this means going for a five minute walk on flat-then I get confused, I can't remember stuff, so it's like I find it really hard to unpick which way round it is"
8	Participant 11, FG3	"Seven months plus in I don't know whether I'm gonna get my brain back […] I'm really, really fearful for the future or whether I'm going to be able to get back to what I want to do and that's like your identity and yourself and who I am as a person is, you know, a big part of me is being a [allied health professional] and if I can't, if I've lost that, I've lost a huge part of me."
9	Participant 9, FG4	"I found myself restating and reiterating many times professionally where I'm at now in terms of cognitive ability and there's only so many times you can do that before I feel like I'm becoming that person, you know and it's a lot easier to do that in the house but I think professionally it's been really hard"

For peer review only

10	Participant 5, FG4	"a few times that I've been out and had an in-depth conversation with somebody that hasn't managed to get used to how I am, they've sort of said to me "you're going round in circles in your conversation" or "you're not making a lot of sense", when I hadn't quite recognised how repetitive I was being until somebody said it back to me. But even so those same people … can't seem to cut me any slack for it, or can't seem to understand how difficult it is, do you know what I mean? [There] just doesn't seem to be the understanding there and I can understand that because it would be beyond my comprehension as well if I hadn't lived it"
11	Participant 5, FG2	"For me it's been going from working at 110% pace to not being able to get out of bed, not being able to work to not see people, to have to cancel plans, the impact on my life has been a massive transition and getting my head around that has been huge. I'm accepting now that I need to take the time off to get better and although that's really difficult and it's meant letting lots of people down, and there's been a complete change in my life, I've managed to get to that place."
12	Participant 7, FG4	"Me and my husband have got a traffic light system now, so green's fine, he can just talk business at me, amber is like can you just keep 'what's the weather'-like kind of conversation, and then red is just stop, I need to just rest, stop all the sensory input coming in. And that seems to be working quite well now, so literally I've got to say amber or red and it's that thing when you're so tired that you can't even articulate that

For peer review only - http://bmjopen.bmj.com/site/about/guidelines.xhtml

		you're so tired and explain. So that really has helped us and I think might stop quite a lot of rows."
13	Participant 5, FG3	"I find it extraordinary difficult-doctors, GP's that I spoke to, I just couldn't seem to put it across at all, they would just sort of think 'well why are you worrying, of course you're ill, you're not thinking properly, it will pass'. I couldn't seem to get across the enormity of how much it's affected me and how many different struggles there'd been. And I think part of that is because my communication has actually been impaired from it"
14	Participant 8, FG1	"I have to say it was when my GP said 'yes, we recognise what you've got as Long Covid and we're treating it like concussion at the moment until we know more about it, and we will recommend you rest and maybe try these drugs', I mean, I almost broke down it was the acknowledgement of the issue. [It] takes away so much of the stress because, we're all [thinking], you know, 'is this really happening, is this just me malingering or do I really have this thing'. And so that was that was a key moment for me"
15	Participant 7, FG 1	"I had a couple of different GPs that I spoke to at the beginning and then I spoke consistently to the same locum GP and she was very good. It was when I was having quite a difficult time trying to go back to work and I was struggling quite a lot psychologically and she was very supportive, she spent a lot of time with me and that consistency was good"

For peer review only - http://bmjopen.bmj.com/site/about/guidelines.xhtml

16	Participant 13, FG2	"I've treated stroke patients who [have] dysphasia and they can't find the right words so they go around the houses to describe something so that you understand what they mean and it felt a bit like that in a way that you know what you want to say but you can't think what that word is because it doesn't come to the forefront of your mind. So you're trying to think of how you can describe it and I thought 'oh gosh, I've turned into one of my stroke patients' because I'm trying to find another suitable word but it's such a struggle though"

For peer review only

For peer review only - http://bmjopen.bmj.com/site/about/guidelines.xhtml

References

1 Office of National Statistics. Technical article: Updated estimates of the prevalence of post-acute symptoms among people with coronavirus (COVID-19) in the UK: 26 April 2020 to 1 August 2021. 2021.

2 Office of National Statistics. Coronavirus and the social impacts of 'long COVID' on people's lives in Great Britain: 7 April to 13 June 2021. 2021.

3 World Health Organization. In the wake of the pandemic: preparing for Long COVID (2021)

4 Ladds E, Rushforth A, Wieringa S, et al. Persistent symptoms after Covid-19: qualitative study of 114 "long Covid" patients and draft quality principles for services, *BMC Health Serv Res* 2020;20:1144 doi:10.1186/s12913-020-06001-y [published Online First: -12-20].

5 NICE guideline. COVID-19 rapid guideline: managing the long-term effects of COVID-19. 2020;2021.

6 Callard F, Perego E. How and why patients made Long Covid, *Social science & medicine (1982)* 2021;268:113426 doi:10.1016/j.socscimed.2020.113426 [published Online First: Jan].

7 Ziauddeen N, Gurdasani D, O'Hara M,E., et al. Characteristics of Long Covid: findings from a social media survey, 2021.

8 Sudre CH, Murray B, Varsavsky T, et al. Attributes and predictors of long COVID, *Nature medicine* 2021:1-6 doi:10.1038/s41591-021-01292-y [published Online First: Mar 10,].

9 Davis HE, Assaf GS, McCorkell L, et al. Characterizing Long COVID in an International Cohort: 7 Months of Symptoms and Their Impact, 2020 doi:10.1101/2020.12.24.20248802.

10 Wu F, Wang A, Liu M, et al. Neutralizing Antibody Responses to SARS-CoV-2 in a COVID-19 Recovered Patient Cohort and Their Implications. 2020.

11 Lan L, Xu D, Ye G, et al. Positive RT-PCR Test Results in Patients Recovered From COVID-19, *JAMA* 2020;323:1502-3 doi:10.1001/jama.2020.2783 [published Online First: April 21,].

12 Tay MZ, Poh CM, Rénia L, et al. The trinity of COVID-19: immunity, inflammation and intervention, *Nature reviews. Immunology* 2020;20:363-74 doi:10.1038/s41577-020-0311-8 [published Online First: Jun].

13 Colafrancesco S, Alessandri C, Conti F, et al. COVID-19 gone bad: A new character in the spectrum of the hyperferritinemic syndrome? *Autoimmunity Reviews* 2020;19:102573 doi:10.1016/j.autrev.2020.102573 [published Online First: July 1,].

For peer review only - http://bmjopen.bmj.com/site/about/guidelines.xhtml

14 Landi F, Gremese E, Bernabei R, et al. Post-COVID-19 global health strategies: the need for an interdisciplinary approach, *Aging clinical and experimental research* 2020;32:1613-20 doi:10.1007/s40520-020-01616-x [published Online First: Aug].

15 Forte G, Favieri F, Tambelli R, et al. COVID-19 Pandemic in the Italian Population:Validation of a Post-Traumatic Stress Disorder Questionnaire and Prevalence of PTSD Symptomatology, *International Journal of Environmental Research and Public Health* 2020 [published Online First: June 10,].

16 Jiang H, Nan J, Lv Z, et al. Psychological impacts of the COVID-19 epidemic on Chinese people: Exposure, post-traumatic stress symptom, and emotion regulation, *Asian Pacific Journal of Tropical Medicine* 2020;13:252 doi:10.4103/1995-7645.281614 [published Online First: 6/1/].

17 Nalbandian A, Sehgal K, Gupta A, et al. Post-acute COVID-19 syndrome, *Nature medicine* 2021;27:601-15 doi:10.1038/s41591-021-01283-z [published Online First: Apr].

18 Taquet M, Geddes JR, Husain M, et al. 6-month neurological and psychiatric outcomes in 236 379 survivors of COVID-19: a retrospective cohort study using electronic health records, *The Lancet. Psychiatry* 2021;8:416-27 doi:10.1016/S2215-0366(21)00084-5 [published Online First: May].

19 Michelen M, Cheng V, Manoharan L, et al. Characterising long term Covid-19: a living systematic review. 2020.

20 Graham EL, Clark JR, Orban ZS, et al. Persistent neurologic symptoms and cognitive dysfunction in non-hospitalized Covid-19 "long haulers", *Annals of Clinical and Translational Neurology* 2021;8:1073-85 doi:https://doi.org/10.1002/acn3.51350.

21 Hampshire A, Trender W, Chamberlain SR, et al. EClinicalMedicine. 2021.

22 Lamontagne SJ, Winters MF, Pizzagalli DA, et al. Post-acute sequelae of COVID-19: Evidence of mood & cognitive impairment, *Brain, Behavior, & Immunity - Health* 2021;17:100347 doi:10.1016/j.bbih.2021.100347 [published Online First: November 1,].

23 Anonymous . Definition of neurocognitive - NCI Dictionary of Cancer Terms - National Cancer Institute. 2011;2021.

24 Kanberg N, Ashton NJ, Andersson L, et al. Neurochemical evidence of astrocytic and neuronal injury commonly found in COVID-19, *Neurology* 2020;95:e1754-9 doi:10.1212/WNL.0000000000010111.

25 de Melo GD, Lazarini F, Levallois S, et al. COVID-19-related anosmia is associated with viral persistence and inflammation in human olfactory epithelium and brain infection in hamsters, *Sci Transl Med* 2021;13 doi:10.1126/scitranslmed.abf8396 [published Online First: -06-02].

26 Mondelli V, Pariante CM. What can neuroimmunology teach us about the symptoms of long-COVID? *Oxford Open Immunology* 2021;2 doi:10.1093/oxfimm/iqab004 [published Online First: January 1,].

For peer review only - http://bmjopen.bmj.com/site/about/guidelines.xhtml

27 Lee M, Perl DP, Nair G, et al. Microvascular Injury in the Brains of Patients with Covid-19, *New England Journal of Medicine* 2021;384:481-3 doi:10.1056/NEJMc2033369 [published Online First: February 4,].

28 Alvarez Bravo G, RamióTorrentà L. Encefalitis anti-NMDA-R secundaria a infección por SARS-CoV-2 Anti–NMDA receptor encephalitis secondary to SARS-CoV-2 infection, *Neurología (Barcelona, English ed.)* doi:10.1016/j.nrleng.2020.07.011.

29 Philippens, Ingrid H. C. H. M., Böszörményi KP, Wubben JA, et al. SARS-CoV-2 causes brain inflammation and induces Lewy body formation in macaques, *bioRxiv* 2021:2021.02.23.432474 doi:10.1101/2021.02.23.432474.

30 Douaud G, Lee S, Alfaro-Almagro F, et al. Brain imaging before and after COVID-19 in UK Biobank, *medRxiv* 2021 doi:10.1101/2021.06.11.21258690 [published Online First: -06-20].

31 Lund LC, Hallas J, Nielsen H, et al. Post-acute effects of SARS-CoV-2 infection in individuals not requiring hospital admission: a Danish population-based cohort study, *The Lancet infectious diseases* 2021;21:1373-82 doi:10.1016/S1473-3099(21)00211-5 [published Online First: Oct].

32 Ladds E, Rushforth A, Wieringa S, et al. Developing services for long COVID: lessons from a study of wounded healers, *Clinical medicine (London, England)* 2021;21:59-65 doi:10.7861/clinmed.2020-0962 [published Online First: Jan].

33 Burton A, Aughterson H, Fancourt D, et al. *'I had no life. I was only existing'*. Factors shaping the mental health and wellbeing of people experiencing long Covid: a qualitative study, *medRxiv* 2021:2021.10.13.21264855 doi:10.1101/2021.10.13.21264855.

34 Chase SE. Narrative Inquiry: Towards theoretical and methodological maturity. In: Denzin N, Lincoln Y, eds. The Sage Handbook of Qualitative Research. London: SAGE 2018.

35 Kamberelis G, Dimitriadis G, Welker A. Focus Group Research and/in Figured Worlds.

36 Glaser BG. The Constant Comparative Method of Qualitative Analysis, *Social Problems* 1965;12:436-45 doi:10.2307/798843.

37 Saunders B, Sim J, Kingstone T, et al. Saturation in qualitative research: exploring its conceptualization and operationalization, *Quality & Quantity* 2018;52:1893-907 doi:10.1007/s11135-017-0574-8.

38 Marlow LL, Faull OK, Finnegan SL, et al. PMC6686955; Breathlessness and the brain: the role of expectation, *Curr Opin Support Palliat Care* 2019;13:200-10 doi:10.1097/SPC.0000000000000441 [published Online First: Sep].

39 Stephan KE, Manjaly ZM, Mathys CD, et al. Allostatic Self-efficacy: A Metacognitive Theory of Dyshomeostasis-Induced Fatigue and Depression, *Front Hum Neurosci* 2016;10 doi:10.3389/fnhum.2016.00550.

For peer review only - http://bmjopen.bmj.com/site/about/guidelines.xhtml
For peer review only

40 Harrison OK, Köchli L, Marino S, et al. Interoception of breathing and its relationship with anxiety, *Neuron* 2021 doi:https://doi.org/10.1016/j.neuron.2021.09.045.

41 May CR, Eton DT, Boehmer K, et al. Rethinking the patient: using Burden of Treatment Theory to understand the changing dynamics of illness, *BMC health services research* 2014;14:281 doi:10.1186/1472-6963-14-281 [published Online First: Jun 26,].

42 Bury M. The sociology of chronic illness: a review of research and prospects, *Sociology of Health & Illness* 1991;13:451-68 doi:10.1111/j.1467-9566.1991.tb00522.x.

43 Corbin J, Strauss A. Managing Chronic Illness at Home: Three Lines of Work, *Qualitative sociology* 1985;8:224-47 doi:10.1007/BF00989485.

44 Frank A. The Wounded Storyteller 1995.

45 Scambler G. Health-related stigma, *Sociology of Health & Illness* 2009;31:441-55 doi:https://doi.org/10.1111/j.1467-9566.2009.01161.x.

46 Bate P, Robert G. Experience-based design: from redesigning the system around the patient to co-designing services with the patient, *BMJ Quality & Safety* 2006;15:307-10 doi:10.1136/qshc.2005.016527 [published Online First: /10/01].

47 Schei E. Doctoring as Leadership: the power to heal, *Perspectives in Biology and Medicine* 2006;49:393-406 doi:10.1353/pbm.2006.0048.

48 Frank A. Just Listening: Narrative and Deep Illness, *Families, Systems, & Health* 1998;16:197-212 doi:10.1037/h0089849 [published Online First: September 1,].

49 Heath I. Following the story: continuity of care in general practice. In: Anonymous . Narrative Based Medicine: dialogue and discourse in clinical practice. Londo: BMJ Books 1998:83-92.

50 Office for National Statistics. Research report on population estimates by ethnic group and religion
. 2019.

51 Goffman E. Stigma: notes on the management of spoiled identity. Englewood Cliffs, N.J.: Prentice-Hall 1963.

52 Scambler G, Hopkins A. Being epileptic: coming to terms with stigma, *Sociology of Health & Illness* 1986;8:26-43 doi:10.1111/1467-9566.ep11346455.

53 Fitzgerald MH, Paterson KA. The hidden disability dilemma for the preservation of self, *Journal of Occupational Science* 1995;2:13-21 doi:10.1080/14427591.1995.9686392 [published Online First: April 1,].

54 Valeras A. "We don't have a box": Understanding Hidden Disability Identity Utilizing Narrative Research Methodology, *Disability Studies Quarterly* 2010;30 doi:10.18061/dsq.v30i3/4.1267 [published Online First: /08/24].

For peer review only

55 Kk O, Am B, C S, et al. Exploring disability from the perspective of adults living with HIV/AIDS: development of a conceptual framework. *Health Qual Life Outcomes* 2008;6:76- doi:10.1186/1477-7525-6-76 [published Online First: /10/04].

56 R C. The patient-physician relationship. Narrative medicine: a model for empathy, reflection, profession, and trust. *JAMA* 2001;286:1897-902 doi:10.1001/jama.286.15.1897 [published Online First: /10/01].

57 Al-Sarraj S, Troakes C, Hanley B, et al. The spectrum of neuropathology in COVID-19, *Neuropathology and Applied Neurobiology* 2021;47:3-16 doi:https://doi.org/10.1111/nan.12667.

58 Tay MZ, Poh CM, Rénia L, et al. The trinity of COVID-19: immunity, inflammation and intervention, *Nature reviews. Immunology* 2020;20:363-74 doi:10.1038/s41577-020-0311-8 [published Online First: Jun].

59 Paniz-Mondolfi A, Bryce C, Grimes Z, et al. PMC7264598; Central nervous system involvement by severe acute respiratory syndrome coronavirus-2 (SARS-CoV-2), *J Med Virol* 2020;92:699-702 doi:10.1002/jmv.25915 [published Online First: Jul].

60 Matschke J, Lütgehetmann M, Hagel C, et al. Neuropathology of patients with COVID-19 in Germany: a post-mortem case series, *The Lancet Neurology* 2020;19:919-29 doi:10.1016/s1474-4422(20)30308-2.

61 Netland J, Meyerholz DK, Moore S, et al. PMC2493326; Severe acute respiratory syndrome coronavirus infection causes neuronal death in the absence of encephalitis in mice transgenic for human ACE2, *J Virol* 2008;82:7264-75 doi:10.1128/JVI.00737-08 [published Online First: Aug].

62 Yong SJ. PMC7874499; Persistent Brainstem Dysfunction in Long-COVID: A Hypothesis, *ACS Chem Neurosci* 2021;12:573-80 doi:10.1021/acschemneuro.0c00793 [published Online First: Feb 17].

63 Tarlov AR. Social determinants of health : The sociobiological translation: Routledge 2002:87-109.

64 Office of National Statistics. Prevalence of ongoing symptoms following coronavirus (COVID-19) infection in the UK: 4 November 2021. 2021.

65 Holingue C, Badillo-Goicoechea E, Riehm KE, et al. Mental Distress during the COVID-19 Pandemic among US Adults without a Pre-existing Mental Health Condition: Findings from American Trend Panel Survey, *Prev Med* 2020;139:106231 doi:10.1016/j.ypmed.2020.106231 [published Online First: -10].

66 Ferrucci R, Dini M, Groppo E, et al. PMC7917789; Long-Lasting Cognitive Abnormalities after COVID-19, *Brain Sci* 2021;11 doi:10.3390/brainsci11020235 [published Online First: Feb 13].

67 Jaywant A, Vanderlind WM, Alexopoulos GS, et al. PMC7884062; Frequency and profile of objective cognitive deficits in hospitalized patients recovering from COVID-19,

For peer review only

Neuropsychopharmacology 2021 doi:10.1038/s41386-021-00978-8 [published Online First: Feb 15].

68 Sheng B, Cheng SK, Lau KK, et al. PMC7135192; The effects of disease severity, use of corticosteroids and social factors on neuropsychiatric complaints in severe acute respiratory syndrome (SARS) patients at acute and convalescent phases, *Eur Psychiatry* 2005;20:236-42 doi:10.1016/j.eurpsy.2004.06.023 [published Online First: May].

69 Hickie I, Davenport T, Wakefield D, et al. PMC1569956; Post-infective and chronic fatigue syndromes precipitated by viral and non-viral pathogens: prospective cohort study, *BMJ* 2006;333:575 doi:10.1136/bmj.38933.585764.AE [published Online First: Sep 16].

70 Cairns V, Godwin J. Post-Lyme borreliosis syndrome: a meta-analysis of reported symptoms, *Int J Epidemiol* 2005;34:1340-5 doi:10.1093/ije/dyi129 [published Online First: Dec].

71 National Institute for Health and Care Excellence. Diagnosis of CFS. 2020;2021.

72 Institute of Medicine. IOM 2015 Diagnostic Criteria | CDC. 2015;2021.

73 Carruthers BM, van de Sande M,I., De Meirleir K,L., et al. Myalgic encephalomyelitis: international consensus criteria, 2011;270:327-38.

74 Ocon AJ. Caught in the thickness of brain fog: exploring the cognitive symptoms of Chronic Fatigue Syndrome, *Front Physiol* 2013;0 doi:10.3389/fphys.2013.00063.

75 Wei J, Hou J, Su B, et al. The Prevalence of Frascati-Criteria-Based HIV-Associated Neurocognitive Disorder (HAND) in HIV-Infected Adults: A Systematic Review and Meta-Analysis, *Front Neurol* 2020;11 doi:10.3389/fneur.2020.581346.

76 Yang AC, Kern F, Losada PM, et al. Dysregulation of brain and choroid plexus cell types in severe COVID-19, *Nature* 2021;595:565-71 doi:10.1038/s41586-021-03710-0 [published Online First: /07].

77 BERGER Z, ALTIERY DE JESUS V, ASSOUMOU SA, et al. Long COVID and Health Inequities: The Role of Primary Care, *The Milbank quarterly* 2021;99:519-41 doi:10.1111/1468-0009.12505 [published Online First: Jun].

78 Greenhalgh T, Knight M, A'Court C, et al. Management of post-acute covid-19 in primary care, *BMJ* 2020;370:m3026 doi:10.1136/bmj.m3026.

79 Kingstone T, Taylor AK, O'Donnell CA, et al. Finding the 'right' GP: a qualitative study of the experiences of people with long-COVID, *BJGP Open* 2020;4:bjgpopen20X101143 doi:10.3399/bjgpopen20X101143.

For peer review only

Consolidated criteria for reporting qualitative studies (COREQ): 32-item checklist

Developed from:
Tong A, Sainsbury P, Craig J. Consolidated criteria for reporting qualitative research (COREQ): a 32-item checklist for interviews and focus groups. *International Journal for Quality in Health Care*. 2007. Volume 19, Number 6: pp. 349 – 357

No. Item	Guide questions/description	Reported on Page #
**Domain 1: Research team and reflexivity**		
Personal Characteristics		
1. Inter viewer/facilitator	EL<br>LH	Methods (10)
2. Credentials	EL – MBBCh MA(Oxon) MPH MRCS MRCGP PGCert Med Ed PGDip Health Research (Academic GP)<br><br>LH – MPH (Reseacrh Assistant)<br><br>CC – MBBChir MA(Cantab) (Academic GP Trainee)<br><br>KP – BM DPhil FRCA (Clinical Professor in Anaesthesia)<br><br>TG – MBBCh MD FRCGP FRCP FFPH MBA (Clinical Professor of General Practice)	Author Details (1)<br>Methods (10)
3. Occupation	EL – Academic General Practitioner<br><br>LH – Research Assistant in primary care (sociology)<br><br>CC – Academic General Practitioner trainee<br><br>KP – Clinical Professor<br><br>TG – Clinical Professor	Author details (1)
4. Gender	Female	Methods (10)
5. Experience and training	EL has undertaken formal courses as part of a PG Diploma in qualitative methodologies and has over 2 years experience conducting focus groups and interviews<br><br>LH holds an MPH and has several years of	Methods (10)

For peer review only

	experience as a qualitative research assistant.<br><br>CC is undertaking a Post Graduate Diploma in health research, which includes courses on qualitative methodologies.	
Relationship with participants		
6. Relationship established	No – participants were selected from a previous sample. EL and LH were part of the research team on that study but they had no further relationships with participants.	N/A
7. Participant knowledge of the interviewer	Participants were given a brief introduction to the study's aims as stated in the document, which were partly motivated in response to feedback from those who had taken part in the earlier, broader study about individuals' lived experiences of long Covid.	N/A
8. Interviewer characteristics	A full conflict of interest statement is disclosed in the document. Participants were aware that EL and LH had been part of an earlier study team exploring the lived experience of long Covid and were aware of their clinical and academic qualifications. Bias was limited by adopting a narrative approach whereby participants were simply invited to tell their story.	Methods (10)
**Domain 2: study design**		
Theoretical framework		
9. Methodological orientation and Theory	Our methodological orientation centered around narrative inquiry and thematic content analysis informed by an underlying predetermined theoretical framework. This also allowed for emergent themes in the data where appropriate.	Methods (10-11)
Participant selection		
10. Sampling	Convenience sampling from a previously defined cohort, which was itself selected from a combination of convenience, purposive, and snowball approaches. We also used additional convenience sampling to extend this initial sub-sample.<br><br>Please see methods for details.	Methods (9-10)
11. Method of approach	Email or social media advertisement.	Methods (9)
12. Sample size	50	Methods (10)
13. Non-participation	Nil. However only 20/50 responded to the follow-up email at 4-6 months	Methods (11), Table 1 (25)

For peer review only

Setting		
14. Setting of data collection	Remotely via videoconference or individual video interview.	Methods (10)
15. Presence of non-participants	No	n/a
16. Description of sample	50 participants with median age 43, all resident in the UK, of whom 42 were female and 36 White.	Methods (11)<br>Results (12-13)<br>Table 1 (25)
Data collection		
17. Interview guide	No – we employed a simple narrative approach that encouraged participants to tell their individual stories and respond to each other. Some simple prompts were used by researchers to elicit further details.	Methods (10)
18. Repeat interviews	No	N/A
19. Audio/visual recording	Yes consented videorecording via Zoom.	Methods (9-10)
20. Field notes	Yes contemporaneous notes were made by researchers and included in the analysis.	Methods (10)
21. Duration	Between 60 and 90 minutes.	Methods (10)
22. Data saturation	Data saturation was achieved, but was not required to determine sample size	Methods (11)
23. Transcripts returned	No. However participants were offered the opportunity to contact the research team at any point to offer clarification/correction/redactions and all participants were invited to a webinar to discuss and further clarify the study findings.	Patient Involvement Statement (12)
**Domain 3: analysis and findings**		
Data analysis		
24. Number of data coders	2	Methods (10-11)
25. Description of the coding tree	No	N/A
26. Derivation of themes	Derived from the data	Methods (10-11)
27. Software	NVivo12	Methods (10)
28. Participant checking	Yes, in the webinar as discussed in 23. Furthermore, a draft of the paper was shared with 2 experts by experience – clinically trained individuals experiencing long Covid with ongoing symptoms of brain fog, who offered further comments and corrections.	Strengths and limitations (20); Patient Involvement Statement (12)
Reporting		
29. Quotations presented	Yes	Table 2 (27-32)
30. Data and findings consistent	Yes	Results (12-16)<br>Table 2 (27-32)
31. Clarity of major themes	Yes	Results (13)
32. Clarity of minor themes	Yes	Results (12-16)

For peer review only

For peer review only
